# Neuronal and glial characterization in the rostrocaudal axis of the human anterior olfactory nucleus: Involvement in Parkinson’s disease

**DOI:** 10.3389/fnana.2022.907373

**Published:** 2022-07-18

**Authors:** Sandra Villar-Conde, Veronica Astillero-Lopez, Melania Gonzalez-Rodriguez, Daniel Saiz-Sanchez, Isabel Ubeda-Banon, Alicia Flores-Cuadrado, Alino Martinez-Marcos

**Affiliations:** Neuroplasticity and Neurodegeneration Laboratory, Regional Center for Biomedical Research, Ciudad Real Medical School, University of Castilla-La Mancha, Ciudad Real, Spain

**Keywords:** olfaction, olfactory bulb, olfactory system, Lewy pathology, stereology

## Abstract

Hyposmia is one of the prodromal symptoms of Parkinson’s disease (PD) and a red flag in clinical diagnosis. Neuropathologically, this sign correlates with α-synuclein involvement in the anterior olfactory nucleus (AON). Neurodegeneration, microgliosis, and astrogliosis in AON are poorly studied, and bulbar AON is the focus of these studies with contradictory results. Additionally, male sex is a risk marker for developing PD, but sexual dimorphism of neural and glial populations in the AON has rarely been considered. The aim of this study was to analyze the density of NeuN, Iba-1, GFAP, and Lewy bodies (LBs), as well as the relationship of these cell type markers with pathology along the rostrocaudal axis of the AON (bulbar, retrobulbar, cortical anterior, and posterior divisions). Cavalieri, optical fractionator, and area fraction fractionator stereological approaches were used for the volume, cell populations and LBs densities, area fraction, and percentage of overlap. Iba-1 and α-syn intensities were measured using ImageJ. In non-PD (NPD) cases, the volume was lower in the AON at the extremes of the rostrocaudal axis than in the intermediate divisions. Cortical anterior AON volume decreased in PD compared with NPD cases. NeuN density decreased rostrocaudally in AON portions in NPD and PD cases. This occurred similarly in Iba-1 but only in PD samples. Iba-1 intensity significantly increased in bulbar AON between PD and NPD. No changes were found in astrocytes. Eight percent of NeuN, 0.1% of Iba-1, and 0.1% of GFAP areas overlapped with LBs area along the AON portions. The data indicate that bulbar AON, which is the most rostral portion in this axis, could play a major role in the pathology. This could be related to the larger area occupied by LBs in these divisions.

## Introduction

Olfactory dysfunction is one of the characteristic manifestations of Parkinson’s disease (PD) (Doty, 2012; Ubeda-Banon et al., 2020; Tolosa et al., 2021). Hyposmia is a prodromal symptom in 96% of patients, and it is more frequent than cardinal motor symptoms such as tremor at rest (Duda, 2010). It could be correlated with neuropathological aggregation in PD Braak stage 1 of α-synuclein (α-syn, i.e., provoking a synucleinopathy) in the olfactory bulb and, more specifically, in the anterior olfactory nucleus (AON) (Daniel and Hawkes, 1992; Del Tredici et al., 2002; Braak et al., 2003, 2004). Male sex is a risk marker for developing PD (Ullah et al., 2019), but sex has been only occasionally included in olfactory bulb studies (Huisman et al., 2008; Flores-Cuadrado et al., 2021), and its involvement remains unexplored in AON.

The human AON comprises a number of rostrocaudal divisions, including bulbar (AONb), retrobulbar (AONrb), and cortical anterior (AONca) and posterior (AONcp) portions (Ubeda-Banon et al., 2020), all of which are preferentially involved *via* α-syn aggregation in PD (Sengoku et al., 2008; Beach et al., 2009; Ubeda-Banon et al., 2010, 2017; Attems et al., 2014; Flores-Cuadrado et al., 2021). The description of different portions of AON based on its functionality and connectivity is far from known.

Parkinson’s disease is characterized by progressive neurodegeneration (Poewe et al., 2017). Recently, neurodegeneration was reported in the human olfactory bulb (Flores-Cuadrado et al., 2021), whereas it has not been reported in other brain areas, such as the hippocampus (Villar-Conde et al., 2021). To date and to the best of our knowledge, only two studies have analyzed the neuronal population of AONb in PD. While one study reported neurodegeneration (Pearce et al., 1995), the other did not find significant differences with respect to the neuronal population (Doorn et al., 2014). Synucleinopathy and neurodegeneration in AONb have previously been associated with each other (Pearce et al., 1995).

Regarding glial cells, microglia and astroglia reportedly have a dual role in facilitating the clearance and progression of synucleinopathy. Particularly in AONb, microglial alterations in PD samples have been studied once (Doorn et al., 2014). Interestingly, α-syn inclusions are abundant in neuronal and glial cells (Stevenson et al., 2020). Studies on the relationship between neurons, microglia and astroglia and α-syn are also inconclusive (Doorn et al., 2014; Stevenson et al., 2020).

Therefore, neuronal death and subsequent glial alterations in AON could be a possible cause of olfactory dysfunction in PD. This study constitutes a novel stereological analysis of the volume and neuronal, microglial, and astroglial populations along the different rostrocaudal divisions of the AON (AONb, AONrb, AONca, and AONcp) in PD and non-PD (NPD) samples, emphasizing neuronal and glial involvement with the aggregation of α-syn Lewy bodies (LBs) and Lewy neurites (LNs) in PD. In addition, sex was included as a factor for the analysis of different parameters in the AON. This study aims to clarify the differential involvement of AON portions along the rostrocaudal axis and its role in the hyposmia associated with PD.

## Materials and methods

**FIGURE 1:**
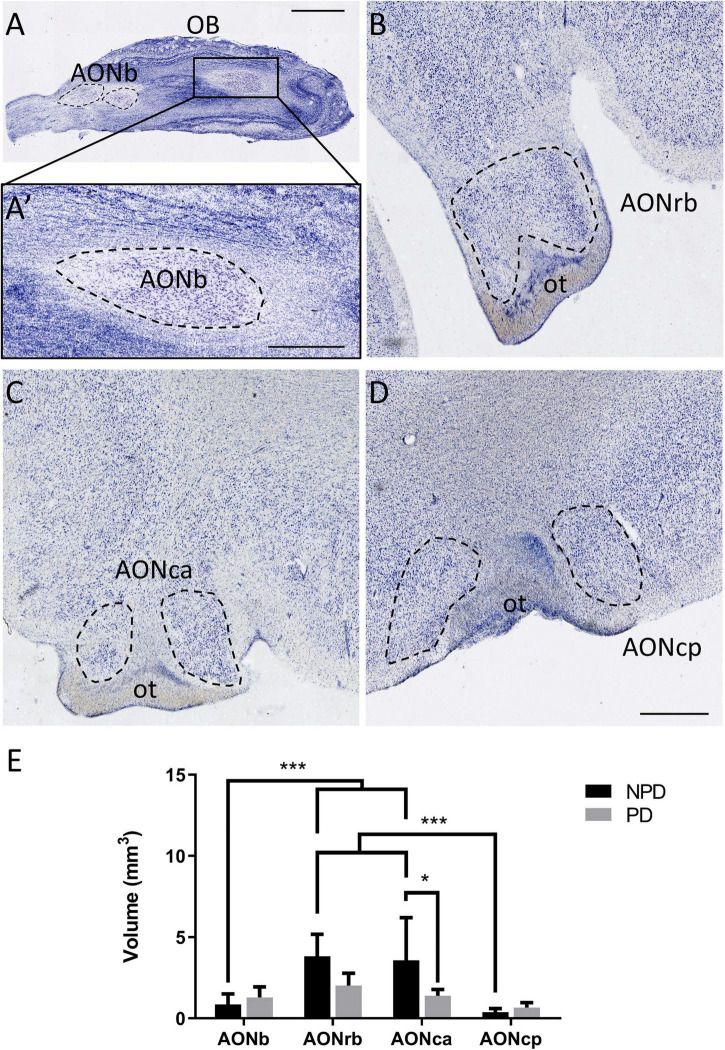
Anterior olfactory nucleus cytoarchitecture and volume. Mosaic reconstruction of Nissl-stained sections of human AON and its volumetric analysis. Horizontal section of the olfactory bulb **(A)**. The frame in panel **(A)** indicates AONb **(A’)**. Coronal section of the cortex in which the dashed line shows AONrb **(B)**, AONca **(C)**, and AONcp **(D)**, and “ot” indicates the olfactory tract. The mean ± SD of volume AON portions in PD and NPD cases **(E)**. *P-value <0.05 and ***P-value <0.001. Scale bar = 2,000 mm **(A)**, 1,000 mm **(A’)**, and 500 mm **(B–D)**.

A total of 41 samples (*n* = 24, PD; *n* = 17, NPD) of AONb, AONrb, AONca, and AONcp were used (Figures 1A–D). The August Pi i Sunyer Biomedical Research Institute (IDIBAPS), the Murcia Region Network Biobank (BIOBANC-MUR), the CIEN Foundation Tissue Biobank (BTCIEN), and the Principality of Asturias Biobank integrated into the National Network of Biobanks of Spain (registration numbers: B.0000575, B.0000859, B.0000741, and B.0000827, respectively) provided the cases and data that they processed following standard operating procedures with the corresponding approval of the ethical and scientific committees (Supplementary Table 1). All experiments were performed in accordance with the Clinical Research Ethics Committee of the University Hospital of Ciudad Real (SAF2016-75768-R and PID2019-108659RB-I00).

The fixed tissue was embedded in a phosphate-buffered solution of 2% dimethyl sulfoxide and 10% glycerol for 48 h and in a phosphate-buffered solution of 2% dimethyl sulfoxide and 20% glycerol for 48 h. Tissue was cut with a freezing microtome into a series of longitudinal sections (50 μm) for AONb and coronal sections (50 μm) for AONrb, AONca, and AONcp (Ubeda-Banon et al., 2017). The thicknesses between sections were 250 μm in AONb, 350 μm in AONrb and AONca, and 150 μm in AONcp. The tissues were Nissl-stained in NPD and PD samples and were used for immunohistochemistry or immunofluorescence in NPD samples and for double immunohistochemistry or double immunofluorescence in PD samples.

For double immunohistochemical procedures, epitopes were unmasked by boiling the tissue in citrate buffer under pressure for 2 min. The tissue was then immersed in 98% formic acid for 3 min and rinsed in 0.01 M phosphate-buffered saline, pH 7.4. The activity of endogenous peroxidases was quenched by adding 1% H_2_O_2_ for 20 min. Sections were incubated for 48 h at 4°C with α-syn antibody and for 2 h at room temperature with a secondary biotinylated antibody (Supplementary Table 2). Subsequently, the avidin–biotin complex (ABC standard, Vector) was applied for 90 min to the sections and then reacted with 0.025% 3,3′-diaminobenzidine, 10% nickel ammonium sulfate, and 30% H_2_O_2_. After rinsing and quenching endogenous peroxidases, the sections were incubated with the blocking buffer for 2 h. The sections were incubated with a NeuN antibody overnight at 4°C and for 2 h with a secondary biotinylated antibody (Supplementary Table 2). The avidin–biotin complex was applied and then reacted with 0.025% 3,3′-diaminobenzidine and 0.1% H_2_O_2_. The sections were mounted onto slides and coverslipped with DPX (BDH, Poole, United Kingdom).

For double immunofluorescence, tissue antigenicity was unmasked as described above. Sections were incubated for 72 h at 4°C with Iba-1 or GFAP and α-syn antibodies and for 2 h with fluorescent secondary antibodies at room temperature (Supplementary Table 2). The sections were counterstained with 0.01% 4′,6-diamidino-2-phenylindole (DAPI) for 5 min and coverslipped with PVA-DABCO (Sigma–Aldrich, St Louis, MO, United States). Although the immunohistochemical assay is more efficient than the immunofluorescence staining of aggregates and fibers, double immunofluorescence was performed against GFAP + α-syn and Iba-1 + α-syn. Intricate labeling of GFAP and Iba-1 markers linked to LNs makes the quantification by immunohistochemistry difficult.

Stereo Investigator software (MBF Bioscience coupled with a Zeiss Axio Imager M2 microscope) was used for stereological analyses. The volume was estimated by Cavalieri’s method (Plan-Neofluar 1x/0.025, Ref. 420300-9900). The optical fractionator method was used to analyze cell populations and LBs density stained with specific markers (neurons, NeuN; microglia, Iba-1; astroglia, GFAP; LBs, α-synuclein) (Plan Apochromat, 63x/1.4, Oil lens, Ref. 420782-9900). The area fraction fractionator method was used to study the area fraction occupied by NeuN, LBs, LNs, and NeuN + LBs overlapping areas (Plan Apochromat, 63x/1.4, Oil lens, Ref. 420782-9900); Iba-1, LBs, LNs, and overlapping areas; and GFAP, LBs, LNs, and overlapping areas (Plan Apochromat, 40X/0.95, Ref. 420660-9970) in PD samples (Flores-Cuadrado et al., 2021; Villar-Conde et al., 2021). The total area fraction of different cell populations was calculated by the sum of markers with and without overlapping, except for AONb data in the NeuN study, which was obtained directly by quantification. To represent the overlapping, the total area fraction occupied by the study marker (e.g., Iba-1) was assumed to constitute 100%, and the percentage corresponding to the marker alone and to the overlap of that marker with others (e.g., LBs or LNs) was calculated.

Iba-1 and α-syn intensities were measured in the different portions of the AON. A total of 147 z-stacks (Zeiss Plan-Apochromat 63x/1.4 Oil DIC M27-oil, Ref. 420782-9900-799) were randomly captured using an LSM 800 confocal microscope (Zeiss, Jena, Germany) coupled with Zen 2.3 software. ImageJ software (National Institutes of Health, Bethesda, MD, United States, 1.47v) was used for processing images following this protocol. Briefly, Iba-1 and α-syn channels were split. In each channel, Z projections were performed, and a threshold was applied to consider specific labeling (85 for Iba-1 and 75 for α-syn). After that, “analyze particles” and “masks” were chosen to obtain the intensity of markers.

For the statistical analysis, GraphPad Prism^®^ v.6 (San Diego, CA, United States) was used. Shapiro–Wilk tests (n < 30) were conducted to analyze the normality of the sample (*P* value > 0.05). Two-tailed *t*-tests and Mann–Whitney tests were used to compare ages and weights in the different studies. Two-way ANOVA with Bonferroni’s *post hoc* test was performed to estimate significant intra- and intergroup differences in PD and NPD samples in volume, cell densities, Iba-1 intensity, and sex. Comparisons were performed using a Kruskal–Wallis test followed by Dunn’s *post hoc* test to estimate the significance of LBs densities and area fractions occupied by LBs, LNs, NeuN, Iba-1, and GFAP. Pearson’s and Spearman’s tests were used for correlation analyses. All data are represented as the mean ± standard deviation (SD). Differences were regarded as statistically significant at *P value < 0.05, ^**^P value < 0.01, and ^***^P value < 0.001.

## Results

### Age and brain weight

Age and brain weight were compared between the study groups of all AON divisions using the *t*-test or the Mann–Whitney test. Significant differences in age were found between PD and NPD cases in AONb for the volume and NeuN density study but not in the rest of the analysis or in the other AON divisions (graphics not shown; for statistical data see Supplementary Table 3.1). There were no correlations between volume and age or NeuN density and age in AONb (data not shown). Brain weights between PD and NPD in the different AON portions and study groups were not significantly different (graphics not shown; for statistical data see Supplementary Table 3.1).

### Volume and estimation of neurons, microglia, astroglia, and Lewy bodies

Volume was analyzed by Cavalieri’s method, and NeuN, Iba-1, GFAP, and LBs densities were analyzed by an optical fractionator (see Supplementary Table 3.2 for statistical data and Supplementary Tables 4–8 for more data about the stereological analysis).

Two-way ANOVA multiple comparison analyses reflected significant differences in volume between PD and NPD samples in AONca. Moreover, a significantly lower volume of AONb and AONcp relative to AONrb and AONca was detected in the NPD group (Figure 1E; Supplementary Tables 3.2, 4). There was no correlation between volume and brain weight (graphics not shown; for statistical data, see Supplementary Table 3.3).

**FIGURE 2:**
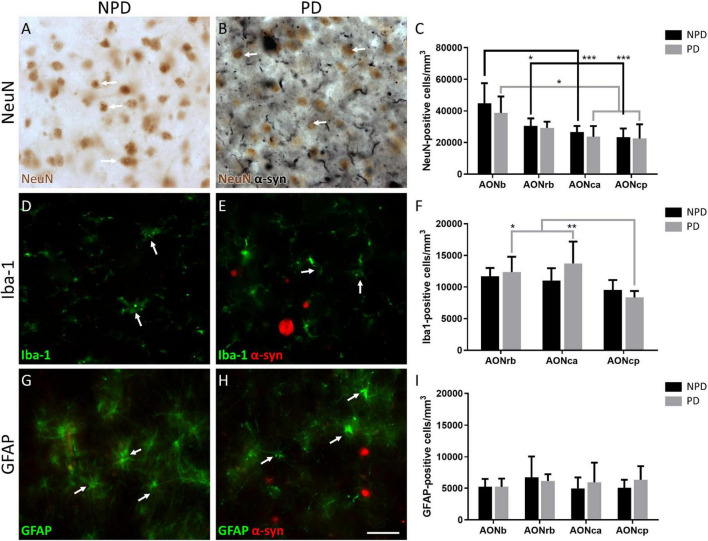
Quantification of neurons and glia. Representation of the labeling in immunohistochemistry of NeuN **(A,B)**, immunofluorescence of Iba-1 **(D,E)**, and GFAP **(G,H)**. Arrows indicate cell types counted NeuN **(A,B)**, Iba-1 **(D,E)**, and GFAP **(G,H)**. The mean ± SD of NeuN **(C)**, Iba-1 **(F)**, and GFAP **(I)** density in NPD and PD samples. *P-value <0.05, **P-value <0.01, and ***P-value <0.001. Scale bar = 25 mm.

**FIGURE 3:**
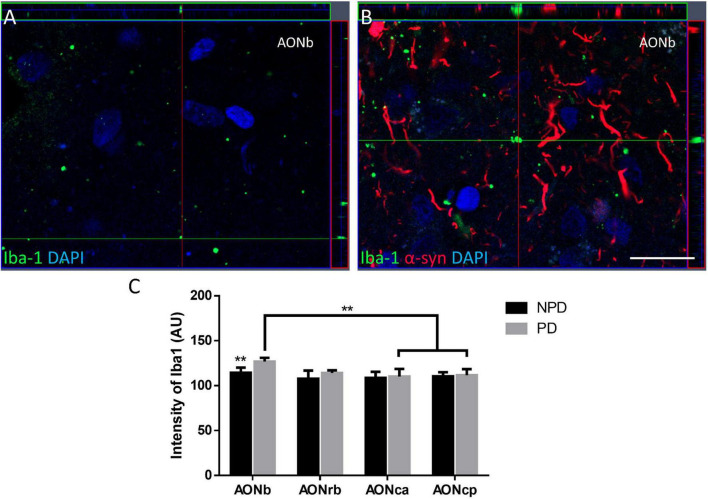
Iba-1 intensity in the AON. Z-stack confocal images of immunofluorescence of Iba-1 and α-syn in AONb in NPD **(A)** and PD **(B)**. The graph depicts the Iba-1 intensity (AU) in AONb, AONrb, AONca, and AON cp **(C)**. **P-value <0.01. Scale bar = 20 mm.

Regarding cell populations, no significant differences were found in NeuN, Iba-1, and GFAP densities between PD and NPD samples in AONb, AONrb, AONca, and AONcp (Figure 2; Supplementary Tables 3.2, 5–7). However, the multiple comparison analyses reflected significant differences in NeuN density in the NPD group and in NeuN and Iba-1 densities in the PD group (Figures 2A–C,E,F). The NeuN population of NPD cases was higher in AONb than in AONrb, AONca, and AONcp, with increasing significance. However, in PD cases, the NeuN population decreased from the AONb to AONca and AONcp with a lower significance when compared with the results of the NPD group (Figures 2A–C; Supplementary Tables 3.2, 5). The population of Iba-1 in the PD group was higher in AONrb and much greater in AONca than in AONcp (Figures 2D–F; Supplementary Tables 3.2, 6). It was not possible to quantify the Iba-1 population in the AONb because the labeling did not reveal well-defined cell somas (Supplementary Figure 1). Instead, Iba-1 intensity was analyzed in all portions of the AON. The intensity of Iba-1 was higher in PD than in NPD cases in the AONb. In addition, the Iba-1 intensity in AONb was increased compared with that in AONca and AONcp in the PD group (Figure 3; for statistical data, see Supplementary Table 3.2). No differences were observed in GFAP density (Figures 2G–I; Supplementary Tables 3.2, 7).

**FIGURE 4:**
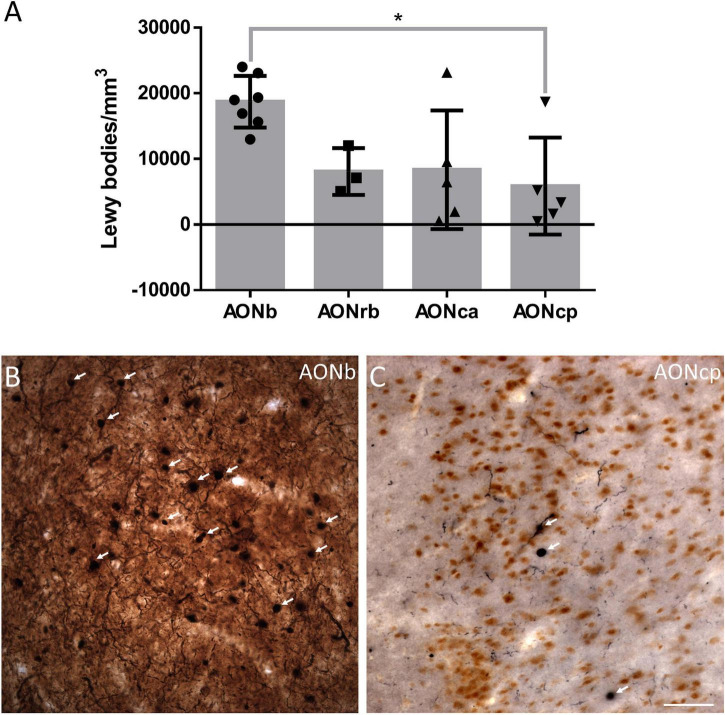
α-synucleinopathy. The mean ± SD of the Lewy bodies density **(A)**. Immunohistochemistry against α-syn in AONb **(B)** and AONcp **(C)**. Arrows point to the LBs. *P-value <0.05. Scale bar = 25 mm.

The density of LBs present in PD cases was significantly higher in AONb (18710 ± 3935 LBs/mm^3^) than in AONcp (8069 ± 3565 LBs/mm^3^) (Kruskal–Wallis statistic: 8.883; *P* value = 0.0309) (Figure 4; Supplementary Table 8). The correlation between NeuN and LBs densities was not significant (graphics not shown; for statistical data, see Supplementary Table 3.4). Moreover, the LBs density did not correlate with the Braak stage (graphics not shown; for statistical data, see Supplementary Table 3.5), whereas there was a significant correlation with PD duration when all AON divisions were considered together (graphics not shown; for statistical data, see Supplementary Table 3.6).

### Area fraction occupied by total NeuN, Iba-1, GFAP, and α-syn (Lewy bodies and Lewy neurites) and by these markers without overlapping

Area fraction fractionator analyses were used to determine the area fraction occupied by total NeuN, Iba-1, GFAP, and α-syn (LBs and LNs) in different AON divisions (for statistical and stereological data, see Supplementary Tables 3.7, 9–11, respectively). Analysis of the total Iba-1 area in AONb was not possible because the labeling did not reveal well-defined cell somas (Supplementary Figure 1). Therefore, AONb was particularly analyzed for total NeuN and GFAP (Supplementary Figures 2A, 4A).

The analysis of different markers showed that the area fraction occupied by total NeuN-positive cells was significantly decreased in AONca and AONcp compared with AONb (Supplementary Figure 2A). Additionally, the total Iba-1 area fraction was greater in AONrb than in AONcp (Supplementary Figure 3A), whereas the area fraction occupied by total GFAP-positive cells was increased in AONca vs. AONcp (Supplementary Figure 4A).

As previously described, α-syn was found in LBs and LNs. Following the rostrocaudal axis of AON divisions, the area fraction occupied by total LBs showed an increasing trend in AONb (quantified at the same time as NeuN in AONrb, AONca, and AONcp and independently by single immunohistochemistry in AONb) (Supplementary Figure 2B). In addition, the area fraction occupied by total LBs was significantly higher in AONrb than in AONcp (quantified at the same time as Iba-1 and GFAP) (Supplementary Figures 3B, 4B). Regarding total LNs, the area fraction of this marker was greater in AONb than in AONca and AONcp (quantified at the same time as NeuN in AONrb, AONca, and AONcp and independently by single immunohistochemistry in AONb) (Supplementary Figure 2C) and was maintained in all AON divisions (quantified at the same time as Iba-1 or GFAP) (Supplementary Figures 3C, 4C).

On the contrary, NeuN, Iba-1, GFAP, LBs, and LNs without overlapping were also counted. The NeuN and Iba-1 area fractions again followed the rostrocaudal axis and were higher in AONrb than in AONcp (Supplementary Figures 2D, 3D). However, no change was observed in the GFAP area fraction (Supplementary Figure 4D). The LBs and LNs area fractions showed a trend of passing through the rostrocaudal axis (Supplementary Figures 2E, 3F, 4E,F), whereas the LBs area fraction was greater in AONrb than in AONcp (Supplementary Figure 3E). Most of the same cases were used for the area fraction studies of Iba-1 and GFAP and their overlap. For this reason, the results of the area fraction of total LBs and total LNs for AONrb, AONca, and AONcp were very similar in both analyses, also serving as an internal control of the technique (Supplementary Figures 3B,C, 4B,C).

### Area fraction occupied by NeuN, Iba-1, or GFAP overlap with α-syn (Lewy bodies and Lewy neurites)

To understand how the pathology affected these cell populations, area fraction fractionator analyses were also used to determine the area fraction occupied by superimposed LBs and NeuN and α-syn (LBs and LNs) and Iba-1 or GFAP in different AON divisions (for statistical and stereological data, see Supplementary Tables 3.7, 9–11, respectively). This study is a continuation of a previous work of the research group (Flores-Cuadrado et al., 2021). This study uses AONb stained immunohistochemically against NeuN and against α-syn, which was used in the previous work. Due to the lack of a series of these cases or of new cases, it was not possible to perform double immunohistochemical tests in AONb against these markers.

The NeuN + LBs area fraction followed the rostrocaudal axis without statistical significance (Supplementary Figure 2F). Consistent with the results above, the Iba-1 + LBs area fraction was significantly higher in AONrb than in AONcp, and no differences were shown in Iba-1 + LNs (Supplementary Figures 3G,H). However, the GFAP + LBs and GFAP + LNs area fractions were maintained along the axis (Supplementary Figures 4G,H).

### Relationship between cell populations and α-syn (Lewy bodies and Lewy neurites)

**FIGURE 5:**
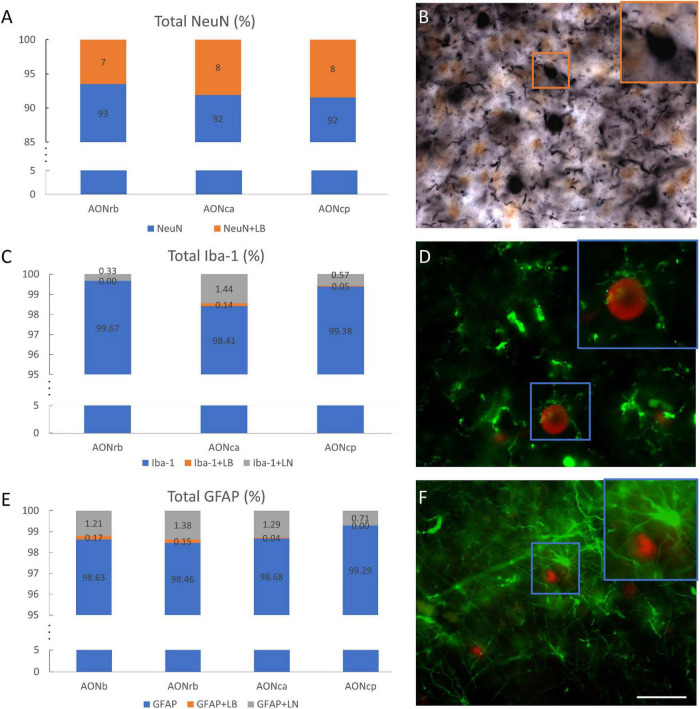
Overlap of cell populations and α-synuclein. Percentage of total NeuN–NeuN without overlapping (blue) and NeuN superimposed with LBs (orange) in different portions of the AON **(A)**. Double immunohistochemistry against NeuN (brown) and α-syn (black). The square indicates the overlap between NeuN and LBs **(B)**. Percentage of total Iba-1-Iba-1 without overlapping (blue), Iba-1 superimposed with LBs (orange) or LNs (gray) in different portions of the AON **(C)**. Double immunofluorescence against Iba-1 (green) and α-syn (red) **(D)**. The square indicates the hug between Iba-1 and LB. Percentage of total GFAP-GFAP without overlapping (blue), GFAP superimposed with LBs (orange) or LNs (gray) in different portions of the AON **(E)**. Double immunofluorescence against GFAP (green) and α-syn (red) **(F)**. Squares indicate the hug between GFAP and LB. Scale bar: 25 μm.

To clarify how cell populations are related to α-syn, different percentages and correlations were calculated. Approximately 7, 8, and 8% of the neuronal area overlapped with the LBs area in AONrb, AONca, and AONcp, respectively (Figures 5A,B). Additionally, approximately 36, 41, and 39% of the LBs area overlapped with the NeuN area in AONrb, AONca, and AONcp, respectively (Supplementary Figure 5A). Therefore, approximately 60% of the LBs area fraction was out of the cells (Figure 5B; Supplementary Figure 5A). The glial population area (Iba-1 and GFAP population) did not overlap with the LBs area by 1% or with the LNs area by 1.5% (Figures 5C–F). Similarly, approximately 0, 5, and 2% of the LBs area and approximately 4, 8, and 5% of the LNs area overlapped with the Iba-1 area in AONrb, AONca, and AONcp, respectively (Supplementary Figures 5B,C), and approximately 17, 7, 2, and 0% of the LBs area and approximately 5, 11, 8, and 5% of the LNs area overlapped with the GFAP area in AONb, AONrb, AONca, and AONcp, respectively (Supplementary Figures 5D,E). Qualitative observations showed that Iba-1 and GFAP exhibited accumulation of LBs, but no colocalization was observed in AON divisions (Figures 5D,F; Supplementary Figure 6).

Considering that the Iba-1 area fraction fractionator cannot be analyzed in AONb, the relationship between the intensity of Iba-1 and the area fraction occupied by the LBs or LNs was examined. No significant correlation in portions or total AON was detected (area fraction _*LBs*_ *P* value = 0.9802; area fraction _*LNs*_ *P* value = 0.0874). Moreover, the intensities of Iba-1 and α-syn were analyzed, and a direct correlation was observed in all AON portions together (*P* value = 0.0002) (graphics not shown; for statistical data see Supplementary Table 3.8).

### Sex analysis

As sex is relevant in neurodegenerative diseases, its involvement was analyzed by two-way ANOVA. There were no inter- or intragroup sex differences (NPD and PD) in volume, Iba-1, GFAP, LBs densities, and Iba-1 intensity (LBs: t_18_: 0.5773, *P* value = 0.5709; for statistical data see Supplementary Table 3.9). However, pathology was a significant source of variation in Iba-1 intensity. NeuN density was significantly influenced by sex (graphics not shown; for statistical data, see Supplementary Table 3.9).

## Discussion

In this report, volume, neurodegeneration, microgliosis and astrogliosis, and α-synucleinopathy involvement were analyzed in the different divisions of the AON in NPD and PD cases. The main goal of the study was to characterize these changes along the key rostrocaudal hubs in the olfactory system and their changes during disease.

Regarding volume studies, the results are controversial. Some studies using MRI did not reveal significant differences in the olfactory bulb volume (Altinayar et al., 2014; Paschen et al., 2015), whereas others did report significant reductions in PD (Wang et al., 2011; Brodoehl et al., 2012). Histologically, a thinner olfactory bulb has been described in PD (Pearce et al., 1995). To the best of our knowledge, our study reports a novel rostrocaudal AON volume characterization. Significant reductions in volume were shown between PD and NPD in AONca. These differences could be due to neuritic loss of specific cell types. Neuritic loss of somatostatin-positive cells has been described in the AONca. These interneurons colocalized with α-syn, especially in the AONca, but not in the other portions of the AON (Ubeda-Banon et al., 2017). Alternatively, this could also be due to the specific functionality/connectivity of this nucleus, which is as yet unknown. Moreover, the most rostral (AONb) and caudal (AONcp) portions presented a significantly lower volume than AONrb and AONca in the NPD group (Figure 1; Supplementary Tables 3.2, 4). These differences disappeared in the PD group, suggesting a possible volume loss in each AON division. Brain weight was not a determinant in these results because there was no correlation with volume (Supplementary Table 3.3).

Early reports point to the olfactory bulb, particularly AONb, as the earliest structures showing Lewy pathology in the Braak stage 1 (Daniel and Hawkes, 1992; Braak et al., 2003; Sengoku et al., 2008; Ubeda-Banon et al., 2010, 2017; Flores-Cuadrado et al., 2021). The specific concentration of α-syn in AONb could trigger neuronal death (Pearce et al., 1995). To date, a single study has correlated a significant loss of neurons stained with cresyl violet with the important presence of LBs in AONb (Pearce et al., 1995). A subsequent study of AONb indicated a lack of differences in the neuronal population labeled with Nissl between PD and NPD (Doorn et al., 2014). Our study analyzed neuronal density with specific markers against NeuN not only in AONb but also in AONrb, AONca, and AONcp (Figure 2; and Supplementary Tables 3.2, 5). We observed that under non-pathological conditions, the NeuN density decreased along the rostrocaudal axis through the different AON portions (Figure 2; Supplementary Tables 3.2, 5). However, the PD group did not present significant differences between AONb and AONrb, but AONb and AONca and AONcp decreased significantly compared with the NPD group (Figure 2; Supplementary Tables 3.2, 5). Regarding synucleinopathy, it has been described a correlation between the accumulation of LBs and neurodegeneration in the AONb (Pearce et al., 1995; Flores-Cuadrado et al., 2021). In agreement, the density of neurons in the AONb in PD was related to the greater amount of LBs present in the AONb (Figures 2A–C, 4; Supplementary Table 3.4). Because age is the main risk factor for PD, PD cases used in the analysis of neuronal density were older than NPD samples in AONb (Supplementary Table 3.1). Despite this fact, no correlations between NeuN density and age were found in AONb (data not shown).

Regarding glia, we first wanted to analyze the population of microglia in AONrb, AONca, and AONcp. We observed that in PD, Iba-1 density was significantly higher in AONrb and AONca than in AONcp (Figure 2; Supplementary Tables 3.2, 6). We hypothesized that Iba-1 density could be increased in AONrb and AONca in the PD group compared with the NPD group; however, the lack of significance could be due to the limited number of cases. These possible differences between PD and NPD in the Iba-1 density of AONrb and AONca could be mainly due to pathology, as we did not observe age differences between the study groups (Supplementary Table 3.1). Furthermore, we hypothesized that the possible increase in microglial density would be greater in the AONb of the PD group than in the NPD group. To corroborate this idea from a different approach rather than stereological quantifications, an intensity analysis of Iba-1 was conducted. Interestingly, the Iba-1 intensity was higher in PD than in NPD in the AONb (Figure 3; Supplementary Table 3.2). In PD, the intensity in AONb was increased compared with that in AONca and AONcp. This is supported by a previous study that indicated an increase in amoeboid microglia marked with CD68 and differentiated from reticular microglia by morphology in PD AONb (Doorn et al., 2014). However, the increase in amoeboid microglia was not correlated with the amount of α-syn, and there was no colocalization between microglia and astroglia with α-syn (Doorn et al., 2014). In agreement with our results, there was no correlation between the intensity of Iba-1 and the area fraction of the LBs or LNs. In contrast, our study revealed that the intensity of Iba-1 was directly correlated with α-syn intensity in all AON divisions in PD cases (Supplementary Table 3.8), similar to the data on olfactory bulb (Flores-Cuadrado et al., 2021). Second, we studied the GFAP population in AONb, AONrb, AONca, and AONcp. Our analysis confirms that GFAP density was maintained as constant in AONb and AONrb and in AONca and AONcp (Figure 2; Supplementary Tables 3.2, 7). In agreement with this result, a previous report showed the GFAP population without finding changes between PD and NPD in AONb (Doorn et al., 2014).

Supporting the data from the optical fractionator probe, the analysis of the area fraction fractionator indicated that the neuronal population occupied a greater area in the rostral portions of the AON than in the more caudal regions (AONca and AONcp). For Iba-1, we found that it occupies a greater area in AONrb than in AONcp. This result was expected considering that the area occupied by the LBs is also greater in the AONrb.

A recent study reported that 8.60% of NeuN presents α-syn in AONb (Stevenson et al., 2020). Our analysis showed very similar data in AONrb, AONca, and AONcp. Approximately 36, 41, and 39% of the LBs area overlapped with 7, 8, and 8% of the total NeuN area in AONrb, AONca, and AONcp, respectively (Figure 5; Supplementary Figure 5 and Supplementary Table 9). In previous reports, LBs were assumed to be inside neurons (Goedert et al., 2013). However, our results showed that 60% of the LBs area was outside neurons. In addition, for the almost zero percentage of overlap of glial area plus LBs area or LNs area, it was qualitatively observed that microglia and astroglia do not normally internalize α-syn but rather surround the accumulations with their projections (Figure 5; Supplementary Figure 6). Our data differ from a previous report on AONb that indicated a 7.78% and a 1.97% overlap between microglia and α-syn and between astrocytes and α-syn, respectively, in AONb (Stevenson et al., 2020).

Male sex is one of the major risk factors for PD. Epidemiological studies show a higher prevalence in men, who have two times the risk of developing the disease compared with women (Ullah et al., 2019). Therefore, it is relevant to analyze sex in relation to the rest of the parameters analyzed in the AON. In this study, inter- or intrasexual differences in volume, LBs, Iba-1, and GFAP densities in AON. In agreement with our results, volume, α-syn aggregates, and GFAP density did not reveal sexual variations in the olfactory bulb (Flores-Cuadrado et al., 2021). On the contrary, a two-way ANOVA demonstrated that sex was significantly associated with NeuN density, without pathology having a major influence. According to olfactory bulb research, neurodegeneration and microgliosis were only significant among PD and NPD male cases (Flores-Cuadrado et al., 2021). To date, this study is the first to analyze the sex factor in AON, although it could be necessary to increase the number of samples to measure in the different portions of AON.

In conclusion, there are variations in the different AON portions along the rostrocaudal axis. AONb, which is the most rostral portion in this axis, could play a major role in the pathology, and AONca is the only portion with a volume decrease in PD compared with NPD. Even though no changes in cell populations have been detected, functional deterioration linked to the involvement of the AON may be due to neuritic impairment (Doorn et al., 2014). Using other approaches, such as proteomics analysis, could provide further insights into these changes (Dammalli et al., 2017; Lachen-Montes et al., 2019).

## Data availability statement

The original contributions presented in this study are included in the article/Supplementary Material, further inquiries can be directed to the corresponding authors.

## Author contributions

IU-B, SV-C, AF-C, AM-M, and DS-S: design. SV-C and IU-B: execution. SV-C and AF-C: analysis. SV-C, AF-C, IU-B, and AM-M: writing. VA-L, MG-R, DS-S, AM-M, IU-B, and AF-C: editing of the final version of the manuscript. All authors contributed to the article and approved the submitted version.

## Conflict of interest

The authors declare that the research was conducted in the absence of any commercial or financial relationships that could be construed as a potential conflict of interest.

## Publisher’s note

All claims expressed in this article are solely those of the authors and do not necessarily represent those of their affiliated organizations, or those of the publisher, the editors and the reviewers. Any product that may be evaluated in this article, or claim that may be made by its manufacturer, is not guaranteed or endorsed by the publisher.
