# Supplementary figures and images for: Neuronal and glial characterization in the rostrocaudal axis of the human anterior olfactory nucleus: Involvement in Parkinson’s disease
Source: Front Neuroanat. 2022 Jul 18;16:907373. doi: 10.3389/fnana.2022.907373 (PMC9339613; doi:10.3389/fnana.2022.907373)

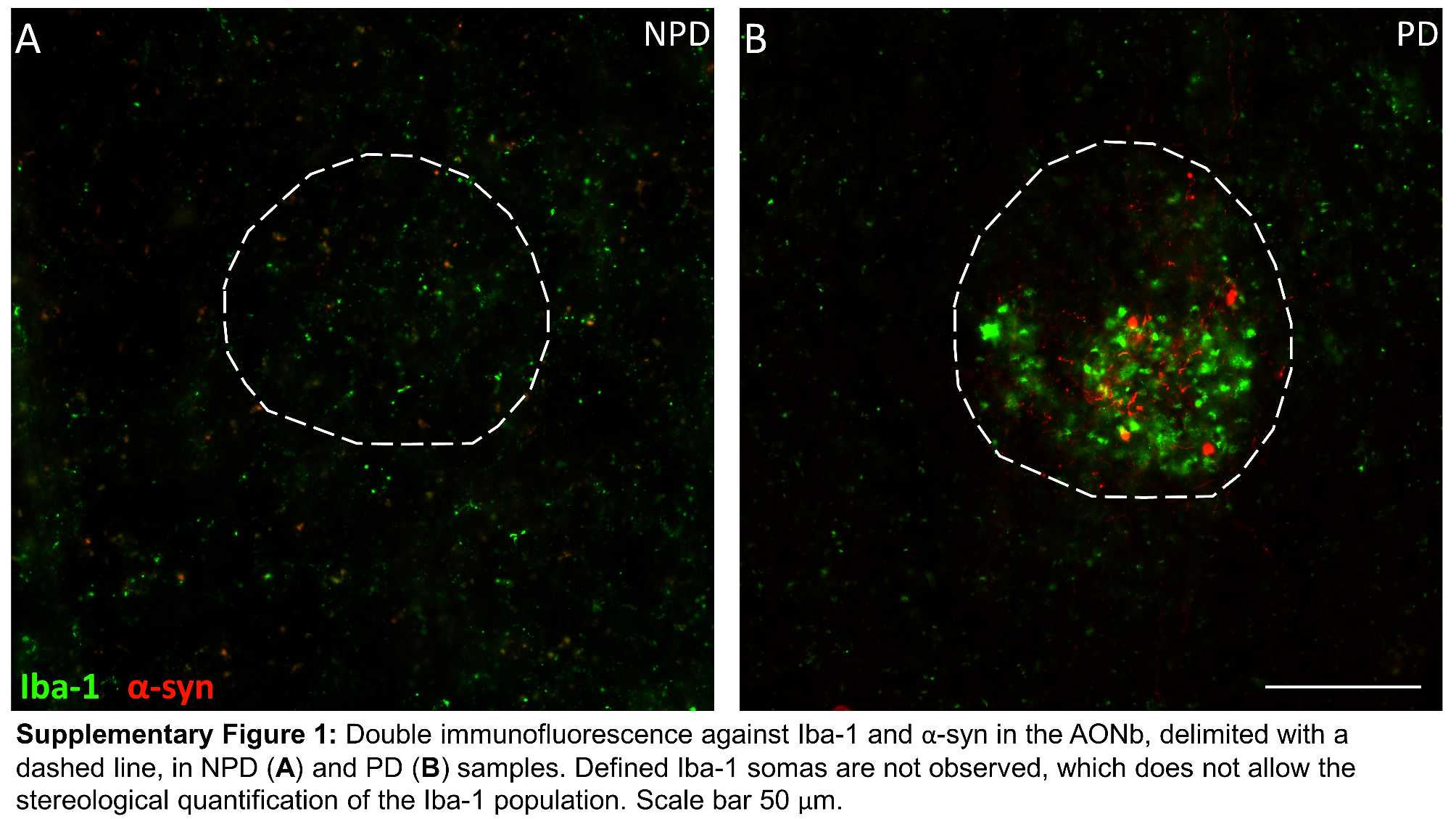

Supplement: Supplementary file 1 [file Image_1.TIF]

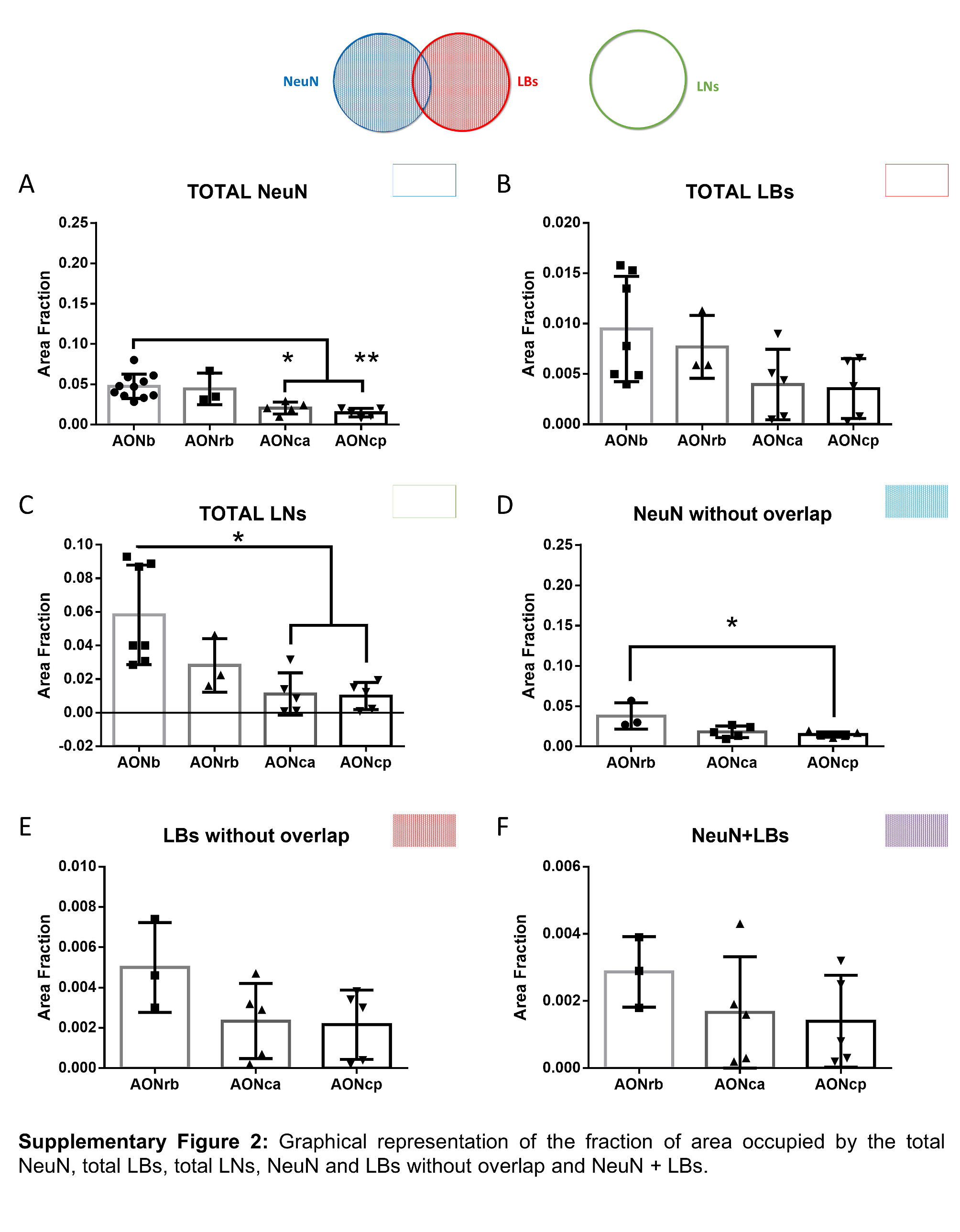

Supplement: Supplementary file 2 [file Image_2.TIF]

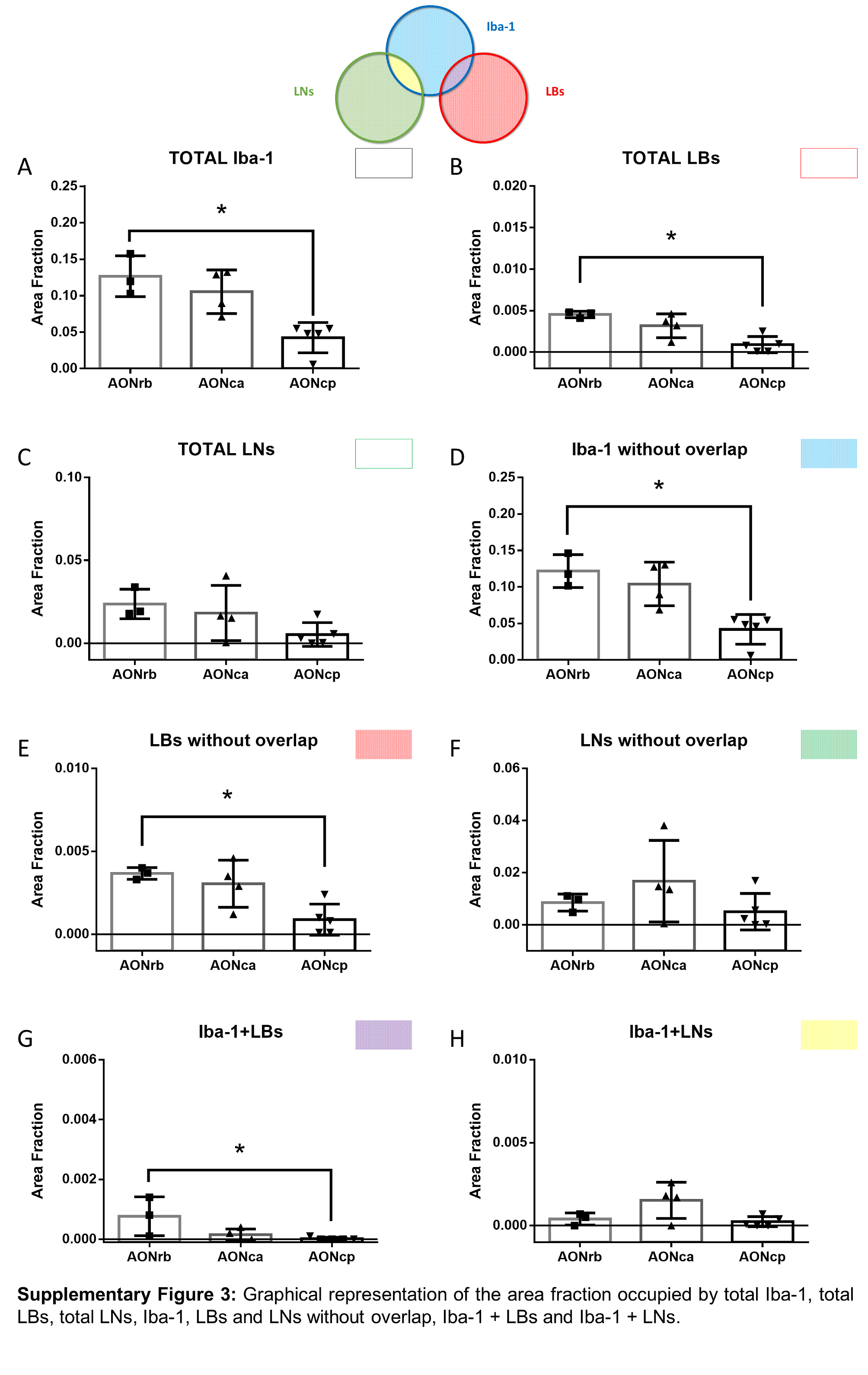

Supplement: Supplementary file 3 [file Image_3.TIF]

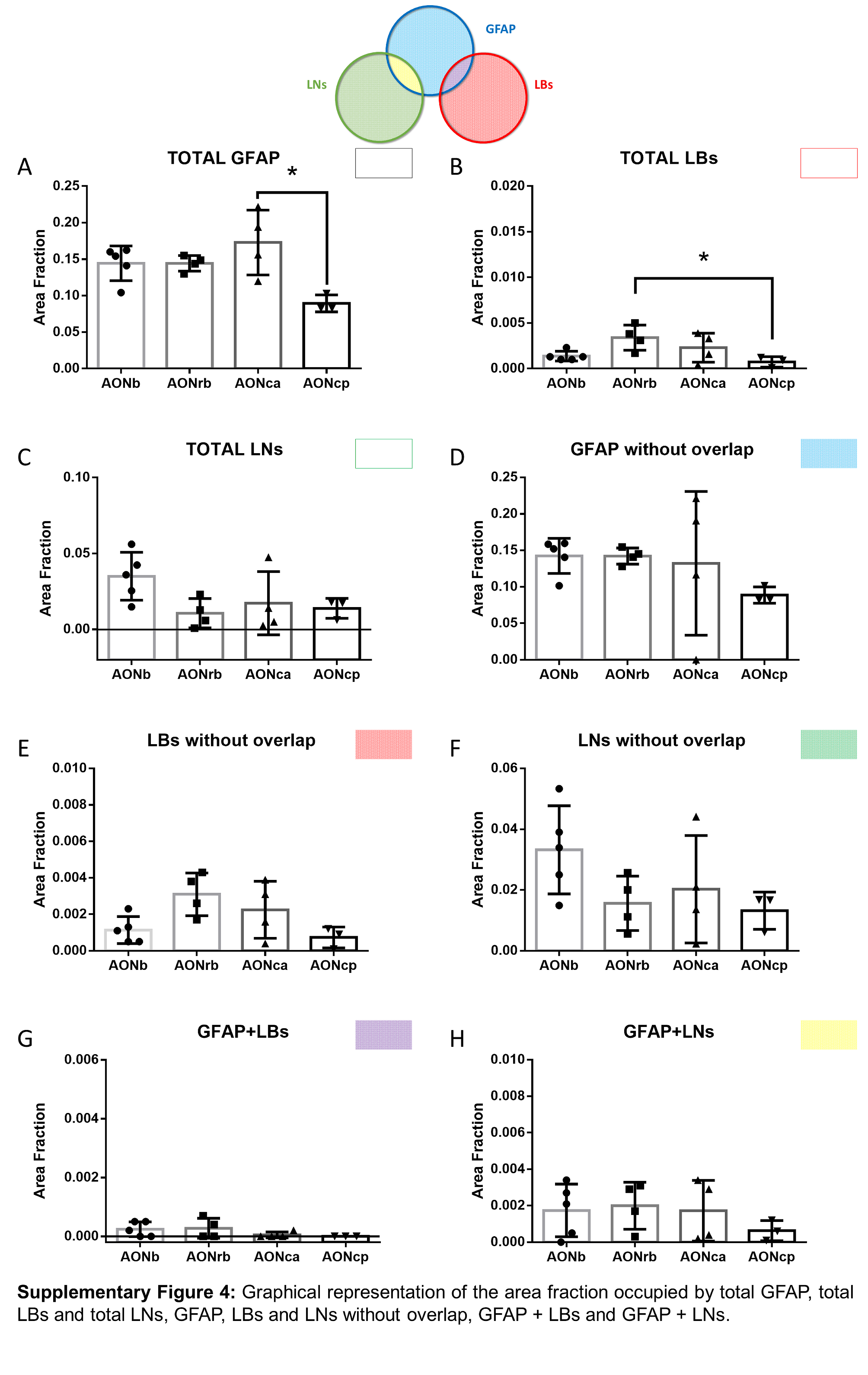

Supplement: Supplementary file 4 [file Image_4.TIF]

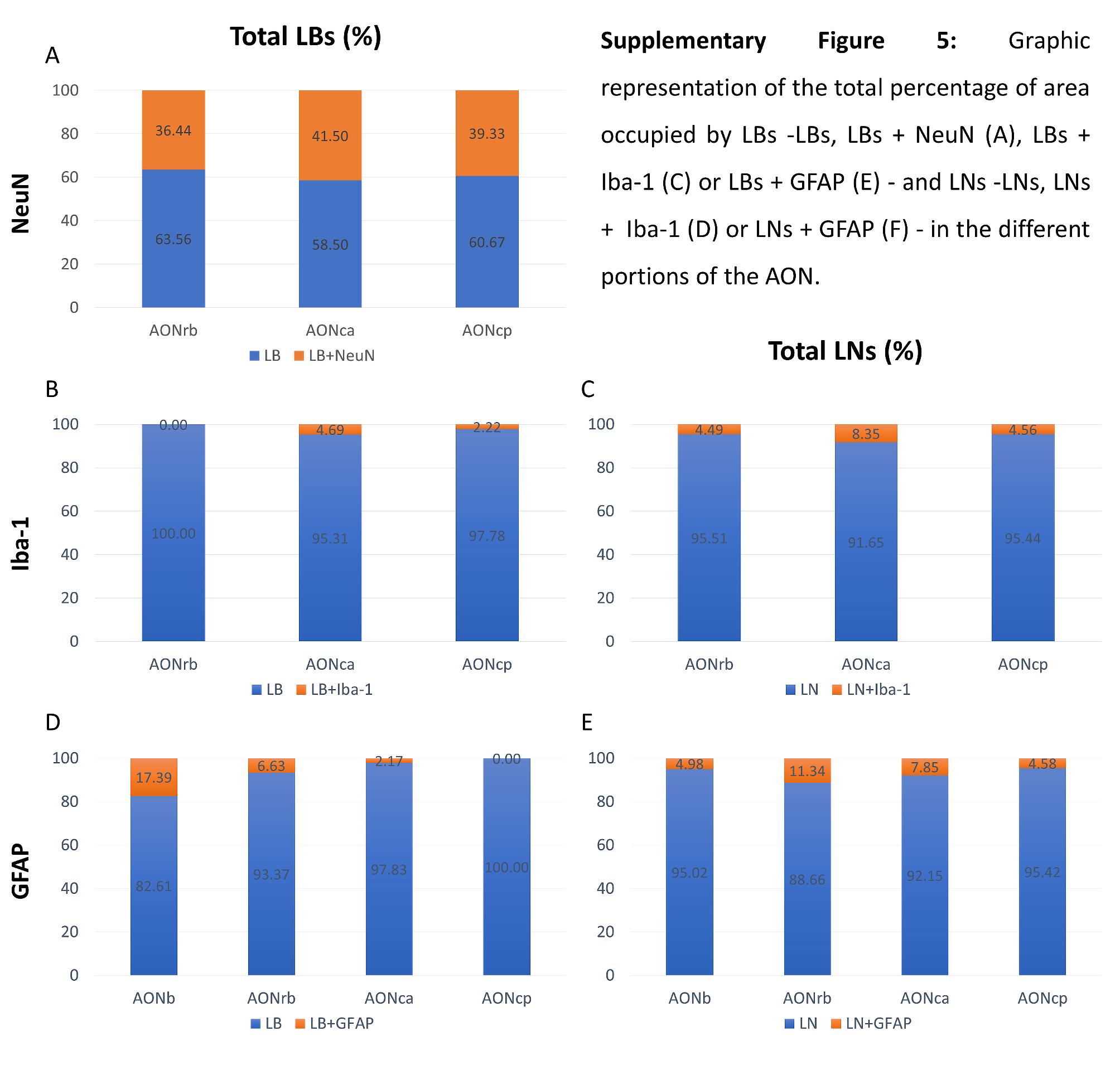

Supplement: Supplementary file 5 [file Image_5.TIF]

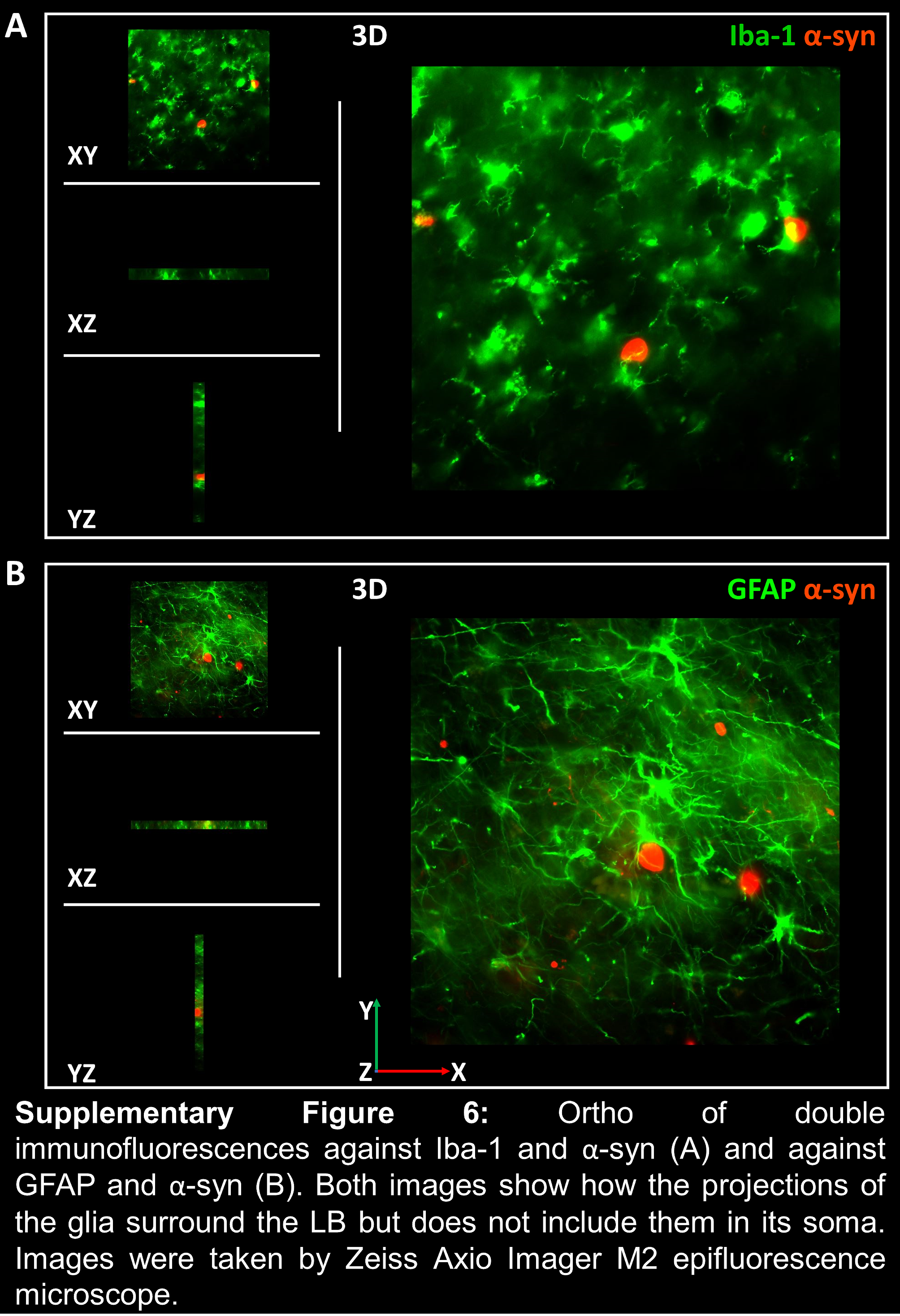

Supplement: Supplementary file 6 [file Image_6.TIF]
