# Supplementary material for: Neuronal and glial characterization in the rostrocaudal axis of the human anterior olfactory nucleus: Involvement in Parkinson’s disease
Source: Front Neuroanat. 2022 Jul 18;16:907373. doi: 10.3389/fnana.2022.907373 (PMC9339613; doi:10.3389/fnana.2022.907373)
Supplement: Supplementary file 7 [file Data_Sheet_1.PDF]

Neuronal and glial characterization in the rostrocaudal axis of the human anterior olfactory nucleus: involvement in Parkinson's disease

Sandra Villar-Conde, Veronica Astillero-Lopez, Melania Gonzalez-Rodriguez, Daniel Saiz-Sanchez, Isabel Ubeda-Banon, Alicia Flores-Cuadrado*, Alino Martinez-Marcos*

Supplementary Table 1. Data of cases used in this study.

Supplementary Table 1.1: Demographic, clinicopathological features and assay of the individuals with PD.

**PD cases**	**Braak stage**	**AON**	**Cavalieri**	**α-syn**	**NeuN**	**NeuN + α-syn**	**Iba-1 + α-syn**	**GFAP + α-syn**	**BH**	**A**	**Sex**	**PMD**	**W**	**D-PD**	**Cause of death**
1	6	b	X		X				R	73	M	1:00	1450	1.5	Cardiorespiratory arrest
2	5	b	X		X				L	82	F	02:00	1300	22	Cardiorespiratory arrest (inmediate), intestinal ischemia (secondary)
3	6	b	X	X	X				L	81	F	12:20	1105	13	Urinary sepsis
4	4	b	X	X	X				L	84	F	04:30	1050	22	Acute myocardial infection
5	4	b	X	X	X				L	81	F	06:30	1010	12	Liver carcinome
6	6	b	X	X	X				L	87	F	07:00	1140	20	Respiratory infection (bronchaspiration)
7	5	b	X	X	X				L	78	M	05:15	1210	9	Respiratory infection (bronchaspiration)
8	5	b	X	X	X				L	75	F	03:45	1095	12	Multiorgan failure
9	6	b	X		X				L	72	M	06:00	1160	15	Advanced cognitive impairment + kindney failure
10	5	b	X	X	X				L	85	M	12:15	1355	11	Respiratory infection (bronchaspiration)
11	4	b					X	X	L	83	M	11:00	1430	23	Acute respiratory failure, recurrent stroke
12	4	b					X	X	L	90	F	05:30	1265	12	Respiratory failure, bronchial aspiration
13	5	b					X	X	L	81	M	07:20	1405	n.a.	Cardiorespiratory failure
14	4	b					X	X	L	83	F	04:00	1060	10	Multiorgan failure, respiratory infection
15	4	b					X	X	L	77	M	12:00	1310	7	Multiorgan failure
16		b	X		X				R	82	M	5:00	n.a.	8	n.a.
17	6	b	X		X				R	65	M	n.a.	1305	9	n.a.
		rb					X	X							
		cp	X			X	X	X							
18	6	rb	X			X	X	X	R	80	M	05:00	1062	n.a.	Bronchial aspiration pneumonia
		ca	X			X	X	X							
19	5	rb	X			X	X	X	R	79	F	06:00	1210	9	Respiratory insufficiency
		ca	X			X	X	X							
20	5	rb	X			X		X	R	70	M	05:00	644	5	Respiratory infection
21	6	ca	X			X	X	X	R	62	M	05:00	1226	>14	n.a.
		cp	X			X	X								
22	4	ca	X			X			R	79	F	05:00	1025	n.a.	n.a.
		cp	X			X	X	X							
23	6	ca	X			X	X	X	R	75	M	03:00	1350	8	Cardiorespiratory arrest
		cp	X			X	X	X							
24	5	cp	X			X	X		R	77	M	05:00	1320	4	Cardiorespiratory arrest

PD: Parkinson's disease, AON: anterior olfactory nucleus, b: bulbar, rb: retrobulbar, ca: cortical anterior, cp: cortical posterior, α-syn: α-synuclein, BH: brain hemisphere, L: left, R: right, A: age (years), M: male, F: female, PMD: post- mortem delayed (hh:mm), W: autopsy brain weight (g), D-PD: PD duration (years), n.a.: not available.

Neuronal and glial characterization in the rostrocaudal axis of the human anterior olfactory nucleus: involvement in Parkinson’s disease
Sandra Villar-Conde, Veronica Astillero-Lopez, Melania Gonzalez-Rodriguez, Daniel Saiz-Sanchez, Isabel Ubeda-Banon, Alicia Flores-Cuadrado*, Alino Martinez-Marcos*

Supplementary Table 1.2: Demographic, clinicopathological features and assay of the individuals with NPD.

NPD cases	AON	Cavalieri	NeuN	Iba-1	GFAP	BH	A	Sex	PMD	W	Cause of death
25	b	X	X			L	73	M	06:10	1030	Respiratory infection (bronchaspiration)
26	b	X	X			R	53	M	05:00	1300	Cardiorespiratory arrest (immediate), intestinal ischemia (secondary)
27	b	X	X			R	81	F	05:00	1100	Multiorgan failure
28	b				X	R	82	M	04:00	800	Respiratory insufficiency
29	b			X	X	R	68	M	04:00	1220	Cardiorespiratory arrest
30	b			X	X	L	78	F	06:00	1075	Piratory heart failure, multiple brain infarctions
31	b				X	L	83	M	13:00	1630	Pratory heart failure, gastroenteritis
32	b			X		R	77	M	10:31	1300	Bronchial aspiration
33	b	X	X			R	58	F	05:00	944	Pneumonia
	ca	X	X	X	X						
34	rb	X	X	X	X	R	43	M	05:00	1412	Septic shock secondary to pneumonia
	ca	X	X	X	X						
	cp	X	X	X	X						
35	b	X	X			R	75	M	04:00	1250	Multiorgan failure
	rb	X	X	X	X						
	ca	X	X	X	X						
36	b	X	X			R	59	F	02:00	1200	Severe acurate pancreatitis (immediate), cardiorespiratory arrest (secundary)
	rb	X	X	X	X						
	ca	X	X	X							
37	b			X	X	R	83	M	04:00	1152	n.a.
	rb	X	X	X	X						
	ca	X	X								
	cp	X	X	X	X						
38	b	X	X			R	62	F	02:00	1050	Cardiorespiratory arrest
	rb	X	X	X	X						
	ca	X	X	X	X						
39	b	X	X			R	78	M	04:00	1100	Respiratory insufficiency
	ca	X	X		X						
	cp	X	X	X	X						
40	b	X				R	58	M	06:00	1500	Acute myocardial infartion
	cp	X	X	X	X						
41	cp	X	X	X	X	R	84	M	03:00	1400	Cardiac arrest

NPD: non-Parkinson's disease, AON: anterior olfactory nucleus, b: bulbar, rb: retrobulbar, ca: cortical anterior, cp: cortical posterior, α-syn: α-synuclein, BH: brain hemisphere, L: left, R: right, A: age (years), M: male, F: female, PMD: post- mortem delayed (hh:mm), W: autopsy brain weight (g), D-PD: PD duration (years), n.a.: not available.

Neuronal and glial characterization in the rostrocaudal axis of the human anterior olfactory nucleus: involvement in Parkinson's disease

Sandra Villar-Conde, Veronica Astillero-Lopez, Melania Gonzalez-Rodriguez, Daniel Saiz-Sanchez, Isabel Ubeda-Banon, Alicia Flores-Cuadrado*, Alino Martinez-Marcos*

Supplementary Table 2. Primary and secondary antibodies used.

Antigen	Company	Catalog Number	Host	Dilution	BB	Incubation	Secondary antibody
NeuN	Abcam (Cambridge, USA)	ab104225	Rabbit polyclonal	1:500	PBS + 0.4 % TX-100 + 10% NHS	4ºC overnight	1:200 Biotinylated horse anti-rabbit IgG (H+L). Vector laboratories (CA, USA).
α-Synuclein	Novocastra (Newcastle, UK)	NCL-L-ASYN	Mouse monoclonal	1:20	PBS + 0.3 % TX-100	4ºC 48 hours	1:200 Biotinylated horse anti-mouse IgG (H+L). Vector laboratories (CA, USA).
Iba-1	Fujifilm WAKO (Neuss, Germany)	019-19741	Rabbit polyclonal	1:2000	TBS+ 0.3% TX-100	4ºC 72 hours	1:200 Alexa Fluor® 488 donkey anti-rabbit. Invitrogen (Massachusetts, USA).
GFAP	Dako (Denmark)	Z0334	Rabbit polyclonal	1:500	TBS+ 0.3% TX-100	4ºC 72 hours	1:200 Alexa Fluor® 488 donkey anti-rabbit. Invitrogen (Massachusetts, USA).
α-Synuclein	Novocastra (Newcastle, UK)	NCL-L-ASYN	Mouse monoclonal	1:20	TBS+ 0.3% TX-100	4ºC 72 hours	1:200 Alexa Fluor® 568 donkey anti-mouse. Invitrogen (Massachusetts, USA).

Neuronal and glial characterization in the rostrocaudal axis of the human anterior olfactory nucleus: involvement in Parkinson's disease

Sandra Villar-Conde, Veronica Astillero-Lopez, Melania Gonzalez-Rodriguez, Daniel Saiz-Sanchez, Isabel Ubeda-Banon, Alicia Flores-Cuadrado*, Alino Martinez-Marcos*

Supplementary Table 3. Statistical data.

Supplementary Table 3.1. Statistical data about age (years) and brain weight (grams) in each analysis.

	**Samples of volume study**				**Samples of NeuN density study**				**Samples of Iba-1 density/intensity study**				**Samples of GFAP density study**				
	**Mean±SD**	**n**	**U/tdf**	**P value**	**Mean±SD**	**n**	**U/tdf**	**P value**	**Mean±SD**	**n**	**U/tdf**	**P value**	**Mean±SD**	**n**	**U/tdf**	**P value**	
**AONb**	NPD:66.33±10.37<br>PD:778.75±6.38	NPD:9<br>PD:12	t_8=3.393	*0.0030	NPD:67.38±10.57<br>PD:78.75±6.384	NPD:8<br>PD:12	t_{18}=3.015	*0.0074	NPD:76.50±6.245<br>PD:82.80±4.712	NPD:4<br>PD:5	U=4.500	0.2222	NPD:78.80±6.380<br>PD:82.80±4.712	NPD:5<br>PD:5	U=9.000	0.5238	**Age (years)**
**AONrb**	NPD:94.40±15.42<br>PD:76.33±5.51	NPD:5<br>PD:3	U=4.000	0.3929	NPD:94.40±15.42<br>PD:76.33±5.51	NPD:5<br>PD:3	U=4.000	0.3929	NPD:64.40±15.42<br>PD:74.67±8.386	NPD:5<br>PD:3	U=4.000	0.3929	NPD:64.40±15.42<br>PD:73.50±7.234	NPD:5<br>PD:4	U=6.000	0.4127	
**AONca**	NPD:65.43±13.96<br>PD:75.00±7.52	NPD:7<br>PD:5	U=9.000	0.1831	NPD:65.43±13.96<br>PD:75.00±7.52	NPD:7<br>PD:5	U=9.000	0.1831	NPD:59.40±11.41<br>PD:74.00±8.287	NPD:5<br>PD:4	U=2.000	0.0635	NPD:63.20±14.10<br>PD:74.00±8.287	NPD:5<br>PD:4	U=4.000	0.1746	
**AONcp**	NPD:69.20±13.96<br>PD:69.75±7.36	NPD:5<br>PD:5	U=11.00	0.8016	NPD:69.20±13.96<br>PD:69.75±7.36	NPD:5<br>PD:5	U=11.00	0.8016	NPD:69.20±18.02<br>PD:71.60±7.603	NPD:5<br>PD:5	U=11.00	0.8016	NPD:69.20±18.02<br>PD:73.00±7.211	NPD:5<br>PD:3	U=7.000	0.8571	
**AONb**	NPD:1240±207.5<br>PD:1199±138.2	NPD:9<br>PD:11	t_8=0.5348	0.5994	NPD:1208±195.8<br>PD:1199±138.2	NPD:8<br>PD:11	t_{17}=0.1172	0.9080	NPD:11.87±95.96<br>PD:1294±147.2	NPD:4<br>PD:5	U=5.000	0.2857	NPD:1175±300.1<br>PD:1294±174.2	NPD:5<br>PD:5	U=8.000	0.4127	**Brain weight (grams)**
**AONrb**	NPD:1213±133.6<br>PD:972.0±293.5	NPD:5<br>PD:3	U=4.000	0.3571	NPD:1213±133.6<br>PD:972.0±293.5	NPD:5<br>PD:3	U=4.000	0.3571	NPD:1213±133.6<br>PD:1192±122.5	NPD:5<br>PD:3	U=7.000	0.9999	NPD:1215±133.5<br>PD:1055±291.8	NPD:5<br>PD:4	U=7.500	0.6032	
**AONca**	NPD:1258±201.6<br>PD:1175±132	NPD:7<br>PD:5	U=13.50	0.5530	NPD:1258±201.6<br>PD:1175±132	NPD:7<br>PD:5	U=13.50	0.5530	NPD:1310±220.2<br>PD:1212±118.0	NPD:5<br>PD:4	U=7.500	0.5952	NPD:1288±237.6<br>PD:1212±118.0	NPD:5<br>PD:4	U=8.000	0.6825	
**AONcp**	NPD:1313±175.8<br>PD:1245±131.3	NPD:5<br>PD:5	U=8.000	0.4127	NPD:1313±175.8<br>PD:1245±131.3	NPD:5<br>PD:5	U=8.000	0.4127	NPD:1313±175.8<br>PD:1245±131.3	NPD:5<br>PD:5	U=8.000	0.4127	NPD:1313±175.8<br>PD:893.3±490.9	NPD:5<br>PD:3	U=2.000	0.1429	

*Indicate significant differences.

Neuronal and glial characterization in the rostrocaudal axis of the human anterior olfactory nucleus: involvement in Parkinson's disease

Sandra Villar-Conde, Veronica Astillero-Lopez, Melania Gonzalez-Rodriguez, Daniel Saiz-Sanchez, Isabel Ubeda-Banon, Alicia Flores-Cuadrado*, Alino Martinez-Marcos*

Supplementary Table 3.2. Statistical data about volume; NeuN, Iba-1 and GFAP density and Iba-1 intensity.

		**Volume**	**NeuN Density**	**Iba-1 Density**	**GFAP Density**	**Iba-1 Intensity**
**AONb**	Mean±SD	NPD:0.8577±0.6486<br>PD:1.2880±0.6501	NPD:45671±12003<br>PD:36457±10359	-<br>-	NPD:5230±1244<br>PD:5230±1297	NPD:114.2±5.9<br>PD:126.8±4.1
	n	NPD:9<br>PD:12	NPD:8<br>PD:12	-<br>-	NPD:5<br>PD:5	NPD:4<br>PD:5
**AONrb**	Mean±SD	NPD:3,8140±1,3670<br>PD:2.0240±0.7581	NPD:30620±4710<br>PD:29299±3932	NPD:11660±1360<br>PD:12365±2417	NPD:6740±3312<br>PD:6156±1085	NPD:107.8±8.9<br>PD:114.1±3.0
	n	NPD:5<br>PD:3	NPD:5<br>PD:3	NPD:5<br>PD:3	NPD:5<br>PD:4	NPD:5<br>PD:3
**AONca**	Mean±SD	NPD:3.5710±2.6300<br>PD:1.3960±0.3839	NPD:26625±3894<br>PD:23653±6771	NPD:11009±1956<br>PD:13733±3450	NPD:4988±1745<br>PD:5948±3133	NPD:108.6±6.7<br>PD:110.1±8.4
	n	NPD:7<br>PD:5	NPD:7<br>PD:5	NPD:5<br>PD:4	NPD:5<br>PD:4	NPD:5<br>PD:4
**AONcp**	Mean±SD	NPD:0.3819±0.2274<br>PD:0.6656±0.6656	NPD:23440±5449<br>PD:22539±8962	NPD:9531±1562<br>PD:8350±1016	NPD:29535±55683<br>PD:6340±2185	NPD:110.4±4.5<br>PD:111.5±6.9
	n	NPD:5<br>PD:5	NPD:5<br>PD:5	NPD:5<br>PD:4	NPD:5<br>PD:3	NPD:5<br>PD:4
	Interaction	F (3, 43) = 3.653<br>P value= 0.0197*	F (3, 36) = 0.2133<br>P value= 0.8865	F (2, 20) = 1.925<br>P value= 0.1720	F (3, 28) = 0.6657<br>P value= 0.5801	F (3, 27) = 1.469<br>P value = 0.2451
	AON type factor	F (3, 43) = 10.17<br>P value< 0.0001*	F (3, 36) = 10.54<br>P value< 0.0001*	F (2, 20) = 7.177<br>P value= 0.0045*	F (3, 28) = 0.7347<br>P value= 0.5401	F (3, 27) = 5.305<br>P value = 0.0053*
	Pathology factor	F (1, 43) = 5.210<br>P value= 0.0275*	F (1, 36) = 1.206<br>P value= 0.2794	F (1, 20) = 0.8501<br>P value= 0.3675	F (1, 28) = 0.6355<br>P value= 0.4321	F (1, 27) = 5.898<br>P value = 0.0221*

* Indicate significant differences.

Neuronal and glial characterization in the rostrocaudal axis of the human anterior olfactory nucleus: involvement in Parkinson's disease

Sandra Villar-Conde, Veronica Astillero-Lopez, Melania Gonzalez-Rodriguez, Daniel Saiz-Sanchez, Isabel Ubeda-Banon, Alicia Flores-Cuadrado*, Alino Martinez-Marcos*

Supplementary Table 3.3. Statistical data about correlation between volume and brain weight.

	**Equation (linear regression)**	**n**	**Test**	**P value**
AONb	NPD: Y = -0.001465X + 2.630<br>PD: Y = 0.0004147X + 0.6812	NPD: 9<br>PD: 11	NPD: Spearman r = -0.3614<br>PD: Pearson r = 0.1038	NPD: 0.3257<br>PD: 0.7613
AONrb	NPD: Y = 0.001679X + 1.779<br>PD: Y = 0.001269X + 0.7900	NPD: 5<br>PD: 3	NPD: Spearman r = 0.1000<br>PD: Spearman r = 0.5000	NPD: 0.9500<br>PD: >0.9999
AONca	NPD: Y = -0.009231X + 15.18<br>PD: Y = 0.0009159X + 0.3222	NPD: 7<br>PD: 5	NPD: Pearson r = -0.7072<br>PD: Spearman r = 0.2000	NPD: 0.0755<br>PD: 0.7833
AONcp	NPD: Y = 0.0003603X – 0.08901<br>PD: Y = 0.0007392X – 0.2524	NPD: 5<br>PD: 5	NPD: Spearman r = 0.1000<br>PD: Spearman r = 0.6669	NPD: 0.9500<br>PD: 0.2667
Total AONs	NPD: Y = -0.003477X + 6.389<br>PD: Y = -0.0004287X + 1.727	NPD: 26<br>PD: 24	NPD: Spearman r = -0.2375<br>PD: Pearson r = -0.1171	NPD: 0.2428<br>PD: 0.5854

Supplementary Table 3.4. Statistical data about correlation between LBs density and NeuN density.

	**Equation (linear regression)**	**n**	**Test**	**P value**
AONb	PD: Y = -0.1794X + 25251	PD: 7	Pearson r = -0.5609	PD: 0.1902
AONrb	PD: Y = 0.8786X - 17673	PD: 3	Spearman r = 1.000	PD: 0.3333
AONca	PD: Y = 0.06936X + 6709	PD: 5	Spearman r = 0.3000	PD: 0.6833
AONcp	PD: Y = 0.1675X + 2087	PD: 5	Spearman r = -0.1000	PD: 0.9500
Total AONs	PD: Y = 0.2608X + 3824	PD: 20	Pearson r = 0.3421	PD: 0.1399

Supplementary Table 3.5. Statistical data about correlation between LBs density and Braak Stage.

	**Equation (linear regression)**	**n**	**Test**	**P value**
AONb	PD: Y = 647.0X + 15475	PD: 7	Pearson r = 0.1343	PD: 0.7741
AONrb	PD: Y = 5911X - 23454	PD: 3	Spearman r = 0.8660	PD: 0.6667
AONca	PD: Y = 3600X - 11088	PD: 5	Spearman r = 0.1118	PD: >0.9999
AONcp	PD: Y = 2861X - 9588	PD: 5	Spearman r = 0.1118	PD: >0.9999
Total AONs	PD: Y = 607.5X + 8122	PD: 20	Pearson r = 0.05844	PD: 0.8067

Neuronal and glial characterization in the rostrocaudal axis of the human anterior olfactory nucleus: involvement in Parkinson's disease
Sandra Villar-Conde, Veronica Astillero-Lopez, Melania Gonzalez-Rodriguez, Daniel Saiz-Sanchez, Isabel Ubeda-Banon, Alicia Flores-Cuadrado*, Alino Martinez-Marcos*

Supplementary Table 3.6. Statistical data about correlation between LBs density and PD duration.

	**Equation (linear regression)**	**n**	**Test**	**P value**
AONb	PD: Y = -116.3X + 20354	PD: 7	Pearson r = 0.1442	PD: 0.7577
AONrb	PD: Y = 515.7X + 2489	PD: 3	Spearman r = Too few pairs	PD: -
AONca	PD: Y = 3437X - 24415	PD: 5	Spearman r = 0.3333	PD: 0.3333
AONcp	PD: Y = 1453X - 5789	PD: 5	Spearman r = 0.4000	PD: 0.7500
Total AONs	PD: Y = 1081X + 667.8	PD: 20	Pearson r = 0.6247	PD: 0.0097*

* Indicate significant differences.

Neuronal and glial characterization in the rostrocaudal axis of the human anterior olfactory nucleus: involvement in Parkinson's disease

Sandra Villar-Conde, Veronica Astillero-Lopez, Melania Gonzalez-Rodriguez, Daniel Saiz-Sanchez, Isabel Ubeda-Banon, Alicia Flores-Cuadrado*, Alino Martinez-Marcos*

Supplementary Table 3.7. Statistical data about area fraction fractionator study.

	AONb		AONrb		AONca		AONcp			
	**Mean±SD**	**n**	**Mean±SD**	**n**	**Mean±SD**	**n**	**Mean±SD**	**n**	**Kruskal-Wallis statistic**	**P value**
**NeuN**	-	-	0.03787±0.01638	3	0.0183±0.007184	5	0.01486±0.003358	5	17.360	0.0006*
**LBs**	-	-	0.0050±0.002227	3	0.00234±0.001864	5	0.00216±0.001723	5	2.594	0.2935
**NeuN+LBs**	-	-	0.002867±0.00105	3	0.00166±0.001659	5	0.0014±0.001366	5	1.865	0.4206
**Total NeuN**	0.04745±0.01514	11	0.04423±0.01962	3	0.02052±0.007245	5	0.01478±0.005350	5	16.810	0.0008*
**Total LBs**	0.009471±0.005226	7	0.0077±0.003118	3	0.00396±0.003495	5	0.00356±0.002960	5	5.165	0.1601
**Total LNs**	0.05827±0.02964	7	0.0282±0.01591	3	0.01116±0.01263	5	0.00994±0.008113	5	12.450	0.0060*
**Iba-1**	-	-	0.1217±0.02266	3	0.1039±0.02991	4	0.04182±0.02046	5	8.256	0.0090*
**LBs**	-	-	0.0014±0.001153	3	0.00305±0.001420	4	0.00088±0.0009418	5	6.130	0.0350*
**LNs**	-	-	0.0085±0.003270	3	0.01673±0.01564	4	0.00502±0.007006	5	2.349	0.3429
**Iba-1+LBs**	-	-	0.0000±0.00000	3	0.00015±0.0001915	4	0.00002±4.472e-005	5	2.684	0.2909
**Iba-1+LNs**	-	-	0.0004±0.0003606	3	0.001525±0.001094	4	0.00024±0.0003050	5	2.889	0.2530
**Total Iba-1**	-	-	0.1267±0.02798	3	0.1055±0.02990	4	0.04242±0.02071	5	8.285	0.0026*
**Total LBs**	-	-	0.004533±0.0003786	3	0.003175±0.001438	4	0.0009±0.0009823	5	8.532	0.0014*
**Total LNs**	-	-	0.0237±0.008861	3	0.01823±0.01665	4	0.00528±0.007149	5	5.308	0.0624
**GFAP**	0.1424±0.02405	5	0.1422±0.01101	4	0.1322±0.09842	4	0.08877±0.01112	3	4.689	0.2015
**LBs**	0.00114±0.0007403	5	0.0031±0.001175	4	0.00225±0.001559	4	0.0007333±0.0005686	3	7.130	0.0517
**LNs**	0.03322±0.01449	5	0.01563±0.008958	4	0.02025±0.01766	4	0.0132±0.006149	3	4.169	0.2564
**GFAP+LBs**	0.00024±0.0002510	5	0.000275±0.0003403	4	0.00005±1.000e-004	4	0.000±0.000	3	3.579	0.3436
**GFAP+LNs**	0.00174±0.001447	5	0.002±0.001291	4	0.001725±0.001660	4	0.0006333±000005508	3	1.453	0.7245
**Total GFAP**	0.1444±0.02390	5	0.1443±0.01069	4	0.173±0.04448	4	0.08943±0.01166	3	8.021	0.0273*
**Total LBs**	0.00138±0.0005357	5	0.0034±0.001378	4	0.0023±0.001598	4	0.0007333±0.0005686	3	7.902	0.0299*
**Total LNs**	0.03496±0.01574	5	0.0107±0.009621	4	0.01723±0.02078	4	0.0139±0.006589	3	5.870	0.1106

*Indicate significant differences.

Supplementary Table 3.8. Statistical data about correlation between Iba-1 intensity and α-syn.

		**Equation (linear regression)**	**n**	**Test**	**P value**
α-syn intensity	AONb	PD: Y = 2.173X – 87.19	PD: 5	Spearman r = 0.9000	PD: 0.0833
	AONrb	PD: Y = 1.516X – 16.88	PD: 3	Spearman r = 1.000	PD: 0.3333
	AONca	PD: Y = -4.522X + 494.7	PD: 4	Spearman r = -0.8000	PD: 0.3333
	AONcp	PD: Y = 0.4535X + 74.03	PD: 4	Spearman r = -0.4000	PD: 0.7500
	Total AONs	PD: Y = 1.222X + 8.353	PD: 16	Pearson r = 0.8020	PD: 0.0002*
Area fraction occupied by LBs	AONb	PD: Y = 1.930e-006X + 0.001127	PD: 5	Spearman r = 0.5798	PD: 0.3333
	AONrb	PD: Y = 7.089e-005X – 0.003553	PD: 3	Spearman r = 0.5000	PD: >0.9999
	AONca	PD: Y = 3.648e-005X – 0.0008413	PD: 4	Spearman r = 0.6000	PD: 0.4167
	AONcp	PD: Y = -1.193e-005X + 0.001831	PD: 4	Spearman r = -0.2108	PD: 0.6667
	Total AONs	PD: Y = -2.667e-005X + 0.005339	PD: 16	Spearman r = 0.007386	PD: 0.9802
Area fraction occupied by LNs	AONb	PD: Y = -0.0004910X + 0.09924	PD: 5	Spearman r = -0.2000	PD: 0.7833
	AONrb	PD: Y = 0.002786X – 0.2941	PD: 3	Spearman r = 0.5000	PD: >0.9999
	AONca	PD: Y = 0.0004820X – 0.03485	PD: 4	Spearman r = 0.0	PD: >0.9999
	AONcp	PD: Y = 0.0002787X – 0.02883	PD: 4	Spearman r = 0.6000	PD: 0.4167
	Total AONs	PD: Y = 0.0006260X – 0.05319	PD: 16	Pearson r = 0.4409	PD: 0.0874

* Indicate significant differences.

Neuronal and glial characterization in the rostrocaudal axis of the human anterior olfactory nucleus: involvement in Parkinson's disease

Sandra Villar-Conde, Veronica Astillero-Lopez, Melania Gonzalez-Rodriguez, Daniel Saiz-Sanchez, Isabel Ubeda-Banon, Alicia Flores-Cuadrado*, Alino Martinez-Marcos*

Supplementary Table 3.9. Statistical data about sex analysis.

			**Volume**	**NeuN Density**	**Iba-1 Density**	**Iba-1 Intensity**	**GFAP Density**
Total AONs	Female	Mean±SD	NPD:2.293±2.346<br>PD:1.506±0.590	NPD:38445±13705<br>PD:33919±9513	NPD:11899±791<br>PD:11149±3378	NPD:112.1±5.2<br>PD:120.4±9.3	NPD:5165±1186<br>PD:5537529
		n	NPD:9<br>PD:10	NPD:9<br>PD:10	NPD:5<br>PD:3	NPD:6<br>PD:5	NPD:5<br>PD:5
Total AONs	Male	Mean±SD	NPD:1.946±2.036<br>PD:1.120±0.590	NPD:29753±9740<br>PD:27810±10881	NPD:10151±1876<br>PD:12549±4592	NPD:109.1±7.2<br>PD:114.6±8.9	NPD:5630±2272<br>PD:5991±2247
		n	NPD:17<br>PD:15	NPD:16<br>PD:15	NPD:10<br>PD:9	NPD:13<br>PD:11	NPD:15<br>PD:11
		Interaction	F (1, 47) = 0.001772<br>P value = 0.9666	F (1, 46) = 0.1671<br>P value = 0.6846	F (1, 23) = 1.357<br>P value = 0.2561	F (1, 31) = 0.2406<br>P value = 0.6273	F (1, 32) = 5.362e-005<br>P value = 0.9942
		Sex factor	F (1, 47) = 0.6261<br>P value = 0.4328	F (1, 46) = 5.487<br>P value = 0.0235*	F (1, 23) = 0.01658<br>P value = 0.8987	F (1, 31) = 2.376<br>P value = 0.1334	F (1, 32) = 0.3743<br>P value = 0.5450
		Pathology factor	F (1, 47) = 3.032<br>P value = 0.0882	F (1, 46) = 1.048<br>P value = 0.3113	F (1, 23) = 0.3718<br>P value = 0.5480	F (1, 3) = 5.843<br>P value = 0.0217*	F (1, 32) = 0.2381<br>P value = 0.6289

* Indicate significant differences.

Neuronal and glial characterization in the rostrocaudal axis of the human anterior olfactory nucleus: involvement in Parkinson's disease

Sandra Villar-Conde, Veronica Astillero-Lopez, Melania Gonzalez-Rodriguez, Daniel Saiz-Sanchez, Isabel Ubeda-Banon, Alicia Flores-Cuadrado*, Alino Martinez-Marcos*

Supplementary Table 4. Volume data.

Supplementary Table 4a. Estimated AONb volume.

Case	Number of Sections	Section Cut Thickness (µm)	Section Evaluation Interval	Grid Size (µm)	Count	Estimated Area (mm²)	Volume Corrected for Over Projection (mm³)	Gundersen Error m=1
1	3	50	5	250	70	4.38	0.98	0.05
2	4	50	5	250	176	11.00	2.50	0.03
3	3	50	5	250	91	5.69	1.22	0.07
4	3	50	5	250	92	5.75	1.25	0.05
5	3	50	5	250	42	2.63	0.59	0.06
6	4	50	5	250	103	6.44	1.44	0.04
7	2	50	5	250	25	1.56	0.34	0.08
8	2	50	5	250	103	6.44	1.42	0.05
9	5	50	5	250	64	4.00	0.92	0.05
10	3	50	5	250	86	5.38	1.22	0.04
16	4	50	5	250	172	10.75	2.50	0.02
17	5	50	5	250	74	4.63	1.08	0.04
25	3	50	5	250	86	5.38	1.21	0.05
26	4	50	5	250	26	1.63	0.37	0.07
27	3	50	5	250	74	4.63	1.06	0.04
33	3	50	5	250	33	2.06	0.46	0.07
35	3	50	5	250	92	5.75	1.29	0.04
36	3	50	5	250	25	1.56	0.36	0.08
38	4	50	5	250	149	9.31	2.19	0.03
39	5	50	5	250	46	2.88	0.67	0.06
40	3	50	5	250	8	0.50	0.11	0.16

*Gray shading shows PD cases.

Supplementary Table 4b. Estimated AONrb volume.

Case	Number of Sections	Section Cut Thickness (µm)	Section Evaluation Interval	Grid Size (µm)	Count	Estimated Area (mm²)	Volume Corrected for Over Projection (mm³)	Gundersen Error m=1
18	3	50	7	250	68	4.25	1.40	0.05
19	4	50	7	250	138	8.63	2.87	0.04
20	2	50	7	250	89	5.56	1.80	0.05
34	4	50	7	250	198	12.38	4.15	0.03
35	4	50	7	250	136	8.50	5.53	0.03
36	4	50	7	250	85	5.31	1.78	0.04
37	4	50	7	250	165	10.31	3.44	0.03
38	4	50	7	250	199	12.44	4.18	0.03

*Gray shading shows PD cases.

Supplementary Table 4c. Estimated AONca volume.

Case	Number of Sections	Section Cut Thickness (µm)	Section Evaluation Interval	Grid Size (µm)	Count	Estimated Area (mm²)	Volume Corrected for Over Projection (mm³)	Gundersen Error m=1
18	4	50	7	250	40	2.50	0.83	0.06
19	4	50	7	250	80	5.00	1.68	0.04
21	4	50	7	250	87	5.44	1.80	0.06
22	4	50	7	250	68	4.25	1.43	0.04
23	3	50	7	250	57	3.56	1.25	0.05
33	3	50	7	250	78	4.88	1.60	0.04
34	4	50	7	250	95	5.94	1.97	0.04
35	4	50	7	250	82	5.13	1.72	0.04
36	4	50	7	250	60	3.75	1.24	0.05
37	4	50	7	250	206	12.88	4.28	0.03
38	4	50	7	250	372	23.25	7.77	0.04
39	2	50	7	250	377	23.56	6.42	0.05

*Gray shading shows PD cases.

Neuronal and glial characterization in the rostrocaudal axis of the human anterior olfactory nucleus: involvement in Parkinson's disease

Sandra Villar-Conde, Veronica Astillero-Lopez, Melania Gonzalez-Rodriguez, Daniel Saiz-Sanchez, Isabel Ubeda-Banon, Alicia Flores-Cuadrado*, Alino Martinez-Marcos*

Supplementary Table 4d. Estimated AONcp volume.

Case	Number of Sections	Section Cut Thickness (µm)	Section Evaluation Interval	Grid Size (µm)	Count	Estimated Area (mm²)	Volume Corrected for Over Projection (mm³)	Gundersen Error m=1
17	3	50	3	250	57	3.56	0.46	0.06
21	2	50	3	250	36	2.25	0.28	0.06
22	4	50	3	250	77	4.81	0.66	0.04
23	4	50	3	250	117	7.31	0.97	0.04
24	4	50	3	250	118	7.38	0.97	0.04
34	3	50	3	200	19	0.76	0.10	0.08
37	4	50	3	250	25	1.56	0.21	0.08
39	4	50	3	250	49	3.06	0.42	0.05
40	4	50	3	250	65	4.06	0.54	0.04
41	4	50	3	250	76	4.75	0.65	0.04

*Gray shading shows PD cases.

Supplementary Table 5: NeuN stereological quantification.

Supplementary Table 5a: Estimated AONb neuron number and density.

Case	Number of Sections	Section Cut Thickness (µm)	Section Evaluation Interval	Disector Height (Z) (µm)	Guard Zone Distance (µm)	Mean Measured Section Thickness (µm)	Counting Frame Area (XY) (µm²)	Sampling Grid Area (XY) (µm²)	Number of Sampling Sites	Total Markers Counted	Estimated Population Using Mean Section Thickness	Measured Volume (mm³)	Gundersen Error m=1	Density (cell/mm³)
1	3	50	5	13	2	18	2500	40000	73	134	14838.39	0.52	0.10	28362.73
2	3	50	5	13	2	19	2500	40000	173	379	44745.95	1.42	0.06	31529.44
3	3	50	5	13	2	16	2500	40000	92	188	18088.19	0.78	0.08	23073.05
4	2	50	5	13	2	16	2500	40000	90	195	18782.86	0.70	0.09	27015.20
5	3	50	5	13	2	16	2500	22500	53	151	8508.41	0.22	0.09	39540.71
6	3	50	5	13	2	18	2500	40000	89	333	36840.31	0.71	0.09	51797.73
7	3	50	5	13	2	16	2500	40000	99	410	40701.52	0.75	0.06	53923.94
8	2	50	5	13	2	16	2500	40000	72	286	28594.35	0.61	0.09	46771.09
9	3	50	5	13	2	16	2500	40000	72	163	15659.57	0.54	0.08	29010.75
10	3	50	5	13	2	18	2500	40000	59	165	18302.31	0.44	0.09	41573.67
16	4	50	5	13	2	16	2500	40000	185	448	43713.59	1.60	0.01	27335.52
17	4	50	5	13	2	16	2500	40000	92	274	27222.88	0.72	0.07	37551.65
25	2	50	5	13	2	16	2500	22500	110	315	17209.29	0.48	0.09	35578.95
26	4	50	5	13	2	18	2500	40000	38	108	11879.98	0.23	0.10	51467.92
27	4	50	5	13	2	18	2500	40000	60	154	17065.76	0.45	0.09	37568.93
33	3	50	5	13	2	16	2500	40000	35	167	15974.00	0.26	0.08	61118.06
35	3	50	5	13	2	18	2500	40000	146	450	25929.14	1.07	0.09	24325.60
36	4	50	5	13	2	16	2500	40000	32	107	10449.43	0.21	0.10	50948.72
38	3	50	5	13	2	17	2500	40000	105	429	44722.17	0.85	0.07	52720.25
39	8	50	5	13	2	18	2500	40000	171	634	68555.64	1.33	0.06	51641.50

*Gray shading shows PD cases.

Neuronal and glial characterization in the rostrocaudal axis of the human anterior olfactory nucleus: involvement in Parkinson's disease
Sandra Villar-Conde, Veronica Astillero-Lopez, Melania Gonzalez-Rodriguez, Daniel Saiz-Sanchez, Isabel Ubeda-Banon, Alicia Flores-Cuadrado*, Alino Martinez-Marcos*

Supplementary Table 5b: Estimated AONrb neuron number and density.

Case	Number of Sections	Section Cut Thickness (µm)	Section Evaluation Interval	Disector Height (Z) (µm)	Guard Zone Distance (µm)	Mean Measured Section Thickness (µm)	Counting Frame Area (XY) (µm²)	Sampling Grid Area (XY) (µm²)	Number of Sampling Sites	Total Markers Counted	Estimated Population Using Mean Section Thickness	Measured Volume (mm³)	Gundersen Error m=1	Density (cell/mm^3)
18	3	50	7	13	2	17	2500	122500	36	100	46101.08	1.39	0.10	33187.97
19	4	50	7	13	2	17	2500	122500	78	200	88723.55	3.02	0.08	29384.89
20	2	50	7	13	2	17	2500	90000	68	151	49765.10	1.97	0.10	25324.59
34	4	50	7	13		18	2500	122500	103	203	96027.24	4.05	0.07	23695.33
35	4	50	7	13	2	16	2500	122500	75	204	84754.55	2.96	0.08	28667.00
36	4	50	7	13		18	2500	122500	55	144	68037.13	1.97	0.09	34478.16
37	4	50	7	13	2	16	2500	122500	96	273	112882.80	3.65	0.07	30949.43
38	4	50	7	13	2	17	2500	122500	115	375	159189.45	4.51	0.06	35308.74

*Gray shading shows PD cases.

Supplementary Table 5c: Estimated AONca neuron number and density.

Case	Number of Sections	Section Cut Thickness (µm)	Section Evaluation Interval	Disector Height (Z) (µm)	Guard Zone Distance (µm)	Mean Measured Section Thickness (µm)	Counting Frame Area (XY) (µm²)	Sampling Grid Area (XY) (µm²)	Number of Sampling Sites	Total Markers Counted	Estimated Population Using Mean Section Thickness	Measured Volume (mm³)	Gundersen Error m=1	Density (cell/mm^3)
18	4	50	7	13	2	18	2500	10000	297	657	25858.82	0.86	0.05	30006.99
19	4	50	7	13	2	18	2500	90000	64	147	51749.12	1.76	0.09	29393.89
21	4	50	7	13	2	16	2500	90000	74	128	39884.11	1.98	0.10	20193.46
22	4	50	7	13	2	17	2500	10000	476	912	34179.27	1.38	0.05	24848.07
23	3	50	7	13	2	16	2500	10000	417	506	17060.68	1.23	0.06	13822.49
33	3	50	7	13	2	16	2500	122500	46	107	46421.81	1.65	0.10	28114.49
34	4	50	7	13	2	18	2500	122500	57	101	48020.54	2.05	0.10	23455.20
35	4	50	7	13	2	18	2500	122500	51	107	50868.77	1.81	0.10	28163.89
36	4	50	7	13	2	16	2500	202500	159	301	40433.88	1.96	0.06	20595.90
37	4	50	7	13	2	18	2500	202500	61	139	109189.80	3.85	0.90	28372.70
38	4	50	7	13	2	16	2500	202500	121	291	206043.94	8.19	0.07	25150.13
39	2	50	7	13	2	16	2500	202500	120	378	267662.44	8.23	0.07	32521.75

*Gray shading shows PD cases.

Neuronal and glial characterization in the rostrocaudal axis of the human anterior olfactory nucleus: involvement in Parkinson's disease

Sandra Villar-Conde, Veronica Astillero-Lopez, Melania Gonzalez-Rodriguez, Daniel Saiz-Sanchez, Isabel Ubeda-Banon, Alicia Flores-Cuadrado*, Alino Martinez-Marcos*

Supplementary Table 5d: Estimated AONcp neuron number and density.

Case	Number of Sections	Section Cut Thickness (µm)	Section Evaluation Interval	Disector Height (Z) (µm)	Guard Zone Distance (µm)	Mean Measured Section Thickness (µm)	Counting Frame Area (XY) (µm²)	Sampling Grid Area (XY) (µm²)	Number of Sampling Sites	Total Markers Counted	Estimated Population Using Mean Section Thickness	Measured Volume (mm³)	Gundersen Error m=1	Density (cell/mm³)
17	3	50	3	13	2	16	2500	40000	108	140	8440.08	0.55	0.10	15429.48
21	2	50	3	13	2	19	2500	22500	118	225	8943.33	0.32	0.08	27639.30
22	4	50	3	13	2	19	2500	40000	128	342	23941.07	0.67	0.06	35833.27
23	4	50	3	13	2	15	2500	40000	174	276	15785.04	0.86	0.06	18358.16
24	4	50	3	13	2	16	2500	22500	369	530	17132.86	1.11	0.06	15434.87
34	3	50	3	13	2	16	2500	10000	95	252	3637.57	0.11	0.07	32098.28
37	4	50	3	13	2	17	2500	22500	82	143	5104.23	0.23	0.10	21960.38
39	4	50	3	13	2	19	2500	40000	81	141	9893.53	0.40	0.09	24884.25
40	4	50	3	13	2	17	2500	40000	120	202	12747.97	0.63	0.07	20215.65
41	4	50	3	13	2	16	2500	40000	141	217	12912.18	0.72	0.07	18042.39

*Gray shading shows PD cases.

Supplementary Table 6: Iba-1 stereological quantification.

Supplementary Table 6a: Estimated AONrb microglia number and density.

Case	Number of Sections	Section Cut Thickness (µm)	Section Evaluation Interval	Disector Height (Z) (µm)	Guard Zone Distance (µm)	Mean Measured Section Thickness (µm)	Counting Frame Area (XY) (µm²)	Sampling Grid Area (XY) (µm²)	Number of Sampling Sites	Total Markers Counted	Estimated Population Using Mean Section Thickness	Measured Volume (mm³)	Gundersen Error m=1	Density (cell/mm^3)
17	2	50	7	13	2	17	2500	10000	117	112	3990.71	0.33	0.10	12262.96
18	3	50	7	13	2	17	2500	22500	188	158	13017.56	1.30	0.09	9999.66
19	2	50	7	13	2	17	2500	90000	83	108	35608.54	2.40	0.10	14831.33
34	4	50	7	13	2	17	2500	160000	118	100	58529.19	5.79	0.10	10104.85
35	3	50	7	13	2	15	2500	62500	95	131	26824.05	1.98	0.09	13527.62
36	3	50	7	13	2	16	2500	22500	171	181	13802.20	1.10	0.08	12509.02
37	4	50	7	13	2	18	2500	75625	146	129	37822.62	3.49	0.09	10852.51
38	4	50	7	13	2	18	2500	90000	128	119	41527.13	3.67	0.10	11308.30

*Gray shading shows PD cases.

Supplementary Table 6b: Estimated AONca microglia number and density.

Case	Number of Sections	Section Cut Thickness (µm)	Section Evaluation Interval	Disector Height (Z) (µm)	Guard Zone Distance (µm)	Mean Measured Section Thickness (µm)	Counting Frame Area (XY) (µm²)	Sampling Grid Area (XY) (µm²)	Number of Sampling Sites	Total Markers Counted	Estimated Population Using Mean Section Thickness	Measured Volume (mm³)	Gundersen Error m=1	Density (cell/mm^3)
18	3	50	7	13	2	18	2500	22500	106	155	13226.82	0.72	0.09	18340.35
19	4	50	7	13	2	16	2500	62500	143	131	28704.64	2.75	0.09	10422.06
21	4	50	7	13	2	17	2500	62500	161	183	42508.39	2.99	0.09	14240.09
23	3	50	7	13	2	17	2500	40000	129	132	19333.85	1.62	0.10	11929.91
33	4	50	7	13	2	19	2500	15625	237	181	11697.21	1.06	0.08	11084.15
34	4	50	7	13	2	16	2500	40000	183	125	17332.18	2.24	0.09	7725.99
35	4	50	7	13	2	17	2500	40000	145	140	20563.69	1.77	0.09	11642.57
36	3	50	7	13	2	16	2500	40000	146	175	23384.19	1.81	0.08	12929.37
38	3	50	7	13	2	17	2500	90000	139	151	49769.79	4.27	0.09	11664.27

*Gray shading shows PD cases.

Neuronal and glial characterization in the rostrocaudal axis of the human anterior olfactory nucleus: involvement in Parkinson's disease

Sandra Villar-Conde, Veronica Astillero-Lopez, Melania Gonzalez-Rodriguez, Daniel Saiz-Sanchez, Isabel Ubeda-Banon, Alicia Flores-Cuadrado*, Alino Martinez-Marcos*

Supplementary Table 6c: Estimated AONcp microglia number and density.

Case	Number of Sections	Section Cut Thickness (µm)	Section Evaluation Interval	Disector Height (Z) (µm)	Guard Zone Distance (µm)	Mean Measured Section Thickness (µm)	Counting Frame Area (XY) (µm²)	Sampling Grid Area (XY) (µm²)	Number of Sampling Sites	Total Markers Counted	Estimated Population Using Mean Section Thickness	Measured Volume (mm³)	Gundersen Error m=1	Density (cell/mm³)
17	2	50	3	13	2	17	2500	15625	154	122	2990.26	0.30	0.10	9808.02
21	3	50	3	13	2	17	2500	22500	108	161	1421.60	0.07	0.09	20959.73
22	4	50	3	13	2	17	2500	62500	144	108	10601.46	1.29	0.10	8194.30
23	3	50	3	13	2	16	2500	62500	213	151	14146.92	1.81	0.09	7830.51
24	2	50	3	13	2	17	2500	10000	210	124	1943.05	0.26	0.10	7565.45
34	4	50	3	13	2	17	2500	22500	153	105	3749.17	0.46	0.10	8229.99
37	4	50	3	13	2	16	2500	15625	176	155	3588.35	0.33	0.08	10998.13
39	2	50	3	13	2	17	2500	22500	192	158	5617.58	0.57	0.08	9910.97
40	3	50	3	13	2	17	2500	10000	290	173	2724.10	0.36	0.08	7581.41
41	4	50	3	13	2	19	2500	62500	140	118	13231.52	1.21	0.10	10935.86

*Gray shading shows PD cases.

Neuronal and glial characterization in the rostrocaudal axis of the human anterior olfactory nucleus: involvement in Parkinson's disease

Sandra Villar-Conde, Veronica Astillero-Lopez, Melania Gonzalez-Rodriguez, Daniel Saiz-Sanchez, Isabel Ubeda-Banon, Alicia Flores-Cuadrado*, Alino Martinez-Marcos*

Supplementary Table 7: GFAP stereological quantification.

Supplementary Table 7a: Estimated AONb astroglia number and density.

Case	Number of Sections	Section Cut Thickness (µm)	Section Evaluation Interval	Disector Height (Z) (µm)	Guard Zone Distance (µm)	Mean Measured Section Thickness (µm)	Counting Frame Area (XY) (µm²)	Sampling Grid Area (XY) (µm²)	Number of Sampling Sites	Total Markers Counted	Estimated Population Using Mean Section Thickness	Measured Volume (mm³)	Gundersen Error m=1	Density (cell/mm³)
11	3	50	5	13	2	17	2500	10000	255	93	2429.00	0.53	0.12	4567.85
12	5	50	5	13	2	17	2500	10000	477	211	5516.17	0.99	0.08	5560.05
13	2	50	5	13	2	17	2500	10000	152	80	2090.53	0.29	0.15	7143.11
14	3	50	5	13	2	17	2500	10000	302	130	3395.76	0.65	0.10	5240.27
15	4	50	5	13	2	17	2500	10000	332	92	2404.45	0.66	0.12	3641.00
37	2	50	5	13	2	17	2500	10000	273	98	2559.62	0.61	0.14	4181.14
28	2	50	5	13	2	17	2500	10000	88	46	1202.84	0.17	0.16	7256.91
29	2	50	5	13	2	17	2500	10000	269	224	3242.82	0.60	0.10	5404.56
30	3	50	5	13	2	17	2500	10000	120	49	1281.74	0.25	0.15	5033.18
31	2	50	5	13	2	17	2500	10000	330	115	2982.32	0.70	0.13	4272.70

*Gray shading shows PD cases.

Supplementary Table 7b: Estimated AONrb astroglia number and density.

Case	Number of Sections	Section Cut Thickness (µm)	Section Evaluation Interval	Disector Height (Z) (µm)	Guard Zone Distance (µm)	Mean Measured Section Thickness (µm)	Counting Frame Area (XY) (µm²)	Sampling Grid Area (XY) (µm²)	Number of Sampling Sites	Total Markers Counted	Estimated Population Using Mean Section Thickness	Measured Volume (mm³)	Gundersen Error m=1	Density (cell/mm³)
17	2	50	7	13	2	16	2500	5625	274	121	2400.39	0.43	0.09	5589.11
18	2	50	7	13	2	17	2500	10000	291	181	6602.94	0.90	0.09	7369.51
19	2	50	7	13	2	16	2500	10000	356	164	5626.53	1.13	0.09	4958.74
20	4	50	7	13	2	18	2500	22500	258	131	11425.62	1.70	0.08	6705.57
34	4	50	7	13	2	17	2500	62500	160	169	39232.98	3.16	0.08	12395.93
35	3	50	7	13	2	17	2500	10000	472	177	6500.93	1.45	0.08	4481.17
36	3	50	7	13	2	16	2500	22500	295	185	14172.37	2.03	0.08	6969.96
37	4	50	7	13	2	17	2500	40000	213	103	13750.00	2.65	0.10	5198.11
38	4	50	7	13	2	16	2500	75625	262	113	28936.79	6.22	0.09	4653.07

*Gray shading shows PD cases.

Sandra Villar-Conde, Veronica Astillero-Lopez, Melania Gonzalez-Rodriguez, Daniel Saiz-Sanchez, Isabel Ubeda-Banon, Alicia Flores-Cuadrado*, Alino Martinez-Marcos*

Supplementary Table 7c: Estimated AONca astroglia number and density.

Case	Number of Sections	Section Cut Thickness (μm)	Section Evaluation Interval	Disector Height (Z) (μm)	Guard Zone Distance (μm)	Mean Measured Section Thickness (μm)	Counting Frame Area (XY) (μm²)	Sampling Grid Area (XY) (μm²)	Number of Sampling Sites	Total Markers Counted	Estimated Population Using Mean Section Thickness	Measured Volume (mm³)	Gundersen Error m=1	Density (cell/mm³)
18	3	50	7	13	2	17	2500	15625	207	173	10049.73	0.99	0.08	10139.07
19	4	50	7	13	2	18	2500	22500	315	158	13777.77	2.16	0.09	6370.63
21	4	50	7	13	2	16	2500	10000	608	230	8123.40	1.86	0.08	4368.97
23	4	50	7	13	2	16	2500	15625	419	108	5906.25	2.03	0.10	2914.34
33	4	50	7	13	2	17	2500	5625	405	172	3492.71	0.64	0.08	5426.11
34	4	50	7	13	2	17	2500	22500	226	134	11831.93	1.52	0.09	7803.16
35	4	50	7	13	2	18	2500	10000	379	130	5039.23	1.12	0.09	4481.71
38	3	50	7	13	2	16	2500	10000	709	229	8006.00	2.14	0.07	3743.43
39	2	50	7	13	2	16	2500	5625	526	157	3075.15	0.88	0.09	3485.18

*Gray shading shows PD cases.

Supplementary Table 7d: Estimated AONcp astroglia number and density.

Case	Number of Sections	Section Cut Thickness (μm)	Section Evaluation Interval	Disector Height (Z) (μm)	Guard Zone Distance (μm)	Mean Measured Section Thickness (μm)	Counting Frame Area (XY) (μm²)	Sampling Grid Area (XY) (μm²)	Number of Sampling Sites	Total Markers Counted	Estimated Population Using Mean Section Thickness	Measured Volume (mm³)	Gundersen Error m=1	Density (cell/mm³)
17	3	50	3	13	2	16	2500	10000	530	212	3192.63	0.69	0.07	4654.49
22	4	50	3	13	2	17	2500	10000	349	154	2373.88	0.43	0.08	5557.19
23	3	50	3	13	2	17	2500	22500	191	143	4913.34	0.56	0.09	8809.59
34	4	50	3	13	2	17	2500	22500	436	256	9039.55	1.30	0.07	6932.81
37	4	50	3	13	2	17	2500	22500	254	102	3596.39	0.74	0.10	4847.51
39	4	50	3	13	2	17	2500	22500	279	131	4626.76	0.82	0.10	5612.43
40	4	50	3	13	2	17	2500	10000	443	147	2304.49	0.52	0.09	4397.71
41	4	50	3	13	2	17	2500	10000	544	163	2535.14	0.69	0.08	3682.99

*Gray shading shows PD cases.

Neuronal and glial characterization in the rostrocaudal axis of the human anterior olfactory nucleus: involvement in Parkinson's disease

Sandra Villar-Conde, Veronica Astillero-Lopez, Melania Gonzalez-Rodriguez, Daniel Saiz-Sanchez, Isabel Ubeda-Banon, Alicia Flores-Cuadrado*, Alino Martinez-Marcos*

Supplementary Table 8: LBs stereological quantification.

Supplementary Table 8a: Estimated AONb LBs number and density.

Case	Number of Sections	Section Cut Thickness (µm)	Section Evaluation Interval	Disector Height (Z) (µm)	Guard Zone Distance (µm)	Mean Measured Section Thickness (µm)	Counting Frame Area (XY) (µm²)	Sampling Grid Area (XY) (µm²)	Number of Sampling Sites	Total Markers Counted	Estimated Population Using Mean Section Thickness	Measured Volume (mm³)	Gundersen Error m=1	Density (cell/mm^3)
3	4	50	5	13	2	17	2500	62500	119	225	36788.83	1.53	0.10	24000.59
4	5	50	5	13	2	17	2500	62500	95	136	22230.76	1.17	0.10	18976.00
5	4	50	5	13	2	17	2500	40000	87	126	13180.48	0.68	0.10	19338.44
6	3	50	5	13	2	17	2500	62500	90	122	19944.97	1.18	0.10	16901.94
7	5	50	5	13	2	17	2500	62500	138	214	38376.80	1.66	0.08	23095.32
8	2	50	5	13	2	17	2500	22500	221	221	13013.55	1.00	0.08	12998.34
10	3	50	5	13	2	17	2500	40000	118	143	14961.60	0.96	0.09	15656.86

*Gray shading shows PD cases.

Supplementary Table 8b: Estimated AONrb LBs number and density.

Case	Number of Sections	Section Cut Thickness (µm)	Section Evaluation Interval	Disector Height (Z) (µm)	Guard Zone Distance (µm)	Mean Measured Section Thickness (µm)	Counting Frame Area (XY) (µm²)	Sampling Grid Area (XY) (µm²)	Number of Sampling Sites	Total Markers Counted	Estimated Population Using Mean Section Thickness	Measured Volume (mm³)	Gundersen Error m=1	Density (cell/mm^3)
18	3	50	7	13	2	17	2500	40000	112	114	16682.38	1.39	0.10	12009.57
19	4	50	7	13	2	17	2500	40000	225	137	20331.15	2.85	0.09	7130.31
20	2	50	7	13	2	17	2500	22500	273	121	9958.47	1.97	0.10	5067.69

*Gray shading shows PD cases.

Supplementary Table 8c: Estimated AONca LBs number and density.

Case	Number of Sections	Section Cut Thickness (µm)	Section Evaluation Interval	Disector Height (Z) (µm)	Guard Zone Distance (µm)	Mean Measured Section Thickness (µm)	Counting Frame Area (XY) (µm²)	Sampling Grid Area (XY) (µm²)	Number of Sampling Sites	Total Markers Counted	Estimated Population Using Mean Section Thickness	Measured Volume (mm³)	Gundersen Error m=1	Density (cell/mm^3)
18	4	50	7	13	2	18	2500	10000	297	141	5575.07	0.86	0.09	6469.40
19	4	50	7	13	2	18	2500	40000	146	106	16816.29	1.76	0.10	9551.78
21	4	50	7	13	2	16	2500	90000	47	147	45804.41	1.98	0.09	23190.93
22	4	50	7	13	2	17	2500	10000	476	73	2735.84	1.38	0.12	1988.94
23	3	50	7	13	2	16	2500	10000	417	20	674.34	1.23	0.23	546.35

*Gray shading shows PD cases.

Neuronal and glial characterization in the rostrocaudal axis of the human anterior olfactory nucleus: involvement in Parkinson's disease

Sandra Villar-Conde, Veronica Astillero-Lopez, Melania Gonzalez-Rodriguez, Daniel Saiz-Sanchez, Isabel Ubeda-Banon, Alicia Flores-Cuadrado*, Alino Martinez-Marcos*

Supplementary Table 8d: Estimated AONcp LBs number and density.

Case	Number of Sections	Section Cut Thickness (µm)	Section Evaluation Interval	Disector Height (Z) (µm)	Guard Zone Distance (µm)	Mean Measured Section Thickness (µm)	Counting Frame Area (XY) (µm²)	Sampling Grid Area (XY) (µm²)	Number of Sampling Sites	Total Markers Counted	Estimated Population Using Mean Section Thickness	Measured Volume (mm³)	Gundersen Error m=1	Density (cell/mm³)
17	3	50	3	13	2	16	2500	10000	431	124	1832.00	0.55	0.10	3349.12
21	2	50	3	13	2	19	2500	22500	118	152	6041.71	0.32	0.10	18671.86
22	4	50	3	13	2	19	2500	10000	518	61	1076.07	0.67	0.13	1610.58
23	4	50	3	13	2	16	2500	10000	673	28	406.32	0.86	0.19	472.55
24	4	50	3	13	2	16	2500	22500	369	179	5786.38	1.11	0.08	5212.91

*Gray shading shows PD cases.

Neuronal and glial characterization in the rostrocaudal axis of the human anterior olfactory nucleus: involvement in Parkinson's disease

Sandra Villar-Conde, Veronica Astillero-Lopez, Melania Gonzalez-Rodriguez, Daniel Saiz-Sanchez, Isabel Ubeda-Banon, Alicia Flores-Cuadrado*, Alino Martinez-Marcos*

Supplementary Table 9. Area fraction.

Supplementary Table 9a. Area fraction occupied by NeuN without overlapping.

	Case	Number of Sections	Section Cut Thickness (µm)	Section Evaluation Interval	Counting Frame Area (XY) (µm²)	Sampling Grid Area (XY) (µm²)	Grid Spacing (µm)	Number of Sampling Sites	Total Markers Counted	Area Sampling Fraction	Area Fraction	Estimated Area (µm²)	Gundersen Error m=1
AONrb	18	3	50	7	40000	160000	20	42	146	0.25	0.0567	233600.00	0.04
	19	4	50	7	40000	90000	20	130	281	0.44	0.0300	252900.00	0.04
	24	2	50	7	40000	62500	20	124	231	0.64	0.0269	16250.00	0.05
AONca	18	4	50	7	40000	62500	15	82	188	0.64	0.0268	66093.80	0.03
	19	4	50	7	40000	62500	10	135	571	0.64	0.0178	89218.80	0.02
	21	4	50	7	40000	62500	10	141	489	0.64	0.0134	76406.30	0.04
	22	4	50	7	40000	62500	10	113	610	0.64	0.0240	95312.50	0.02
	23	3	50	7	40000	62500	10	99	213	0.64	0.0095	33281.30	0.03
AONcp	17	3	50	3	40000	62500	10	98	303	0.64	0.0136	47343.80	0.02
	21	2	50	3	40000	62500	10	60	237	0.64	0.0169	37031.30	0.05
	22	4	50	3	40000	62500	10	118	557	0.64	0.0195	87031.30	0.02
	23	4	50	3	40000	62500	10	185	464	0.64	0.0109	72500.00	0.03
	24	4	50	3	40000	62500	10	630	221	0.64	0.0134	98437.50	0.03

Supplementary Table 9b. Area fraction occupied by LBs without overlapping.

	Case	Number of Sections	Section Cut Thickness (µm)	Section Evaluation Interval	Counting Frame Area (XY) (µm²)	Sampling Grid Area (XY) (µm²)	Grid Spacing (µm)	Number of Sampling Sites	Total Markers Counted	Area Sampling Fraction	Area Fraction	Estimated Area (µm²)	Gundersen Error m=1
AONrb	18	3	50	7	40000	160000	20	42	19	0.25	0.0074	30400.00	0.10
	19	4	50	7	40000	90000	20	130	42	0.44	0.0046	37800.00	0.09
	24	2	50	7	40000	62500	20	124	26	0.64	0.0030	16250.00	0.09
AONca	18	4	50	7	40000	62500	15	82	20	0.64	0.0029	7031.25	0.10
	19	4	50	7	40000	62500	10	135	102	0.64	0.0032	15937.50	0.03
	21	4	50	7	40000	62500	10	141	172	0.64	0.0047	26875.00	0.04
	22	4	50	7	40000	62500	10	113	17	0.64	0.0007	2656.25	0.12
	23	3	50	7	40000	62500	10	99	4	0.64	0.0002	625.00	0.35
AONcp	17	3	50	3	40000	62500	10	98	67	0.64	0.0030	10468.80	0.05
	21	2	50	3	40000	62500	10	60	53	0.64	0.0038	8281.25	0.06
	22	4	50	3	40000	62500	10	118	12	0.64	0.0004	1875.00	0.14
	23	4	50	3	40000	62500	10	185	7	0.64	0.0002	1093.75	0.22
	24	4	50	3	40000	62500	10	630	159	0.64	0.0034	24843.80	0.04

Neuronal and glial characterization in the rostrocaudal axis of the human anterior olfactory nucleus: involvement in Parkinson's disease

Sandra Villar-Conde, Veronica Astillero-Lopez, Melania Gonzalez-Rodriguez, Daniel Saiz-Sanchez, Isabel Ubeda-Banon, Alicia Flores-Cuadrado*, Alino Martinez-Marcos*

Supplementary Table 9c. Area fraction occupied by NeuN overlapping with LBs.

	Case	Number of Sections	Section Cut Thickness (µm)	Section Evaluation Interval	Counting Frame Area (XY) (µm²)	Sampling Grid Area (XY) (µm²)	Grid Spacing (µm)	Number of Sampling Sites	Total Markers Counted	Area Sampling Fraction	Area Fraction	Estimated Area (µm²)	Gundersen Error m=1
AONrb	18	3	50	7	40000	160000	20	42	10	0.25	0.0039	16000.00	0.13
	19	4	50	7	40000	90000	20	130	17	0.44	0.0018	15300.00	0.12
	24	2	50	7	40000	62500	20	124	25	0.64	0.0029	15625.00	0.08
AONca	18	4	50	7	40000	62500	15	82	11	0.64	0.0016	3867.19	0.16
	19	4	50	7	40000	62500	10	135	62	0.64	0.0019	9687.50	0.05
	21	4	50	7	40000	62500	10	141	156	0.64	0.0043	24375.00	0.15
	22	4	50	7	40000	62500	10	113	4	0.64	0.0002	625.00	0.34
	23	3	50	7	40000	62500	10	99	7	0.64	0.0003	1093.75	0.24
AONcp	17	3	50	3	40000	62500	10	98	17	0.64	0.0008	2656.25	0.10
	21	2	50	3	40000	62500	10	60	35	0.64	0.0025	5468.75	0.06
	22	4	50	3	40000	62500	10	118	10	0.64	0.0003	1562.50	0.16
	23	4	50	3	40000	62500	10	185	7	0.64	0.0002	1093.75	0.22
	24	4	50	3	40000	62500	10	630	151	0.64	0.0032	23593.80	0.05

Neuronal and glial characterization in the rostrocaudal axis of the human anterior olfactory nucleus: involvement in Parkinson's disease

Sandra Villar-Conde, Veronica Astillero-Lopez, Melania Gonzalez-Rodriguez, Daniel Saiz-Sanchez, Isabel Ubeda-Banon, Alicia Flores-Cuadrado*, Alino Martinez-Marcos*

Supplementary Table 9d. Area fraction occupied by total NeuN.

	Case	Number of Sections	Section Cut Thickness (µm)	Section Evaluation Interval	Counting Frame Area (XY) (µm²)	Sampling Grid Area (XY) (µm²)	Grid Spacing (µm)	Number of Sampling Sites	Total Markers Counted	Area Sampling Fraction	Area Fraction	Estimated Area (µm²)	Gundersen Error m=1
AONb	1	3	50	5	22500	90000	10	46	395	0.25	0.0801	158000.00	0.06
	2	3	50	5	22500	40000	10	237	1273	0.56	0.0402	226311.00	0.04
	3	4	50	5	22500	90000	10	76	320	0.25	0.0363	128000.00	0.04
	4	2	50	5	22500	90000	10	54	332	0.25	0.0470	132800.00	0.03
	5	3	50	5	22500	90000	10	26	130	0.25	0.0609	52000.00	0.04
	7	3	50	5	22500	90000	10	68	403	0.25	0.0588	161200.00	0.02
	8	2	50	5	22500	90000	10	42	337	0.25	0.0535	134800.00	0.11
	10	3	50	5	22500	90000	10	43	109	0.25	0.0333	43600.00	0.05
	9	3	50	5	22500	90000	10	48	157	0.25	0.0283	62800.00	0.03
	22	4	50	5	22500	90000	10	117	553	0.25	0.0358	221200.00	0.04
	17	4	50	5	22500	90000	10	72	340	0.25	0.0478	136000.00	0.03
AONrb	18	3	50	7	40000	160000	20	42	172	0.25	0.0668	-	-
	19	4	50	7	40000	90000	20	130	319	0.44	0.0347	-	-
	24	2	50	7	40000	62500	20	124	268	0.64	0.0312	-	-
AONca	18	4	50	7	40000	62500	15	82	205	0.64	0.0293	-	-
	19	4	50	7	40000	62500	10	135	661	0.64	0.0206	-	-
	21	4	50	7	40000	62500	10	141	679	0.64	0.0186	-	-
	22	4	50	7	40000	62500	10	113	616	0.64	0.0243	-	-
	23	3	50	7	40000	62500	10	99	220	0.64	0.0098	-	-
AONcp	17	3	50	3	40000	62500	10	98	327	0.64	0.0147	-	-
	21	2	50	3	40000	62500	10	60	283	0.64	0.0202	-	-
	22	4	50	3	40000	62500	10	118	570	0.64	0.0199	-	-
	23	4	50	3	40000	62500	10	630	382	0.64	0.0081	-	-
	24	4	50	3	40000	62500	10	185	471	0.64	0.0110	-	-

Supplementary Table 9e. Area fraction occupied by total LBs.

	Case	Number of Sections	Section Cut Thickness (µm)	Section Evaluation Interval	Counting Frame Area (XY) (µm²)	Sampling Grid Area (XY) (µm²)	Grid Spacing (µm)	Number of Sampling Sites	Total Markers Counted	Area Sampling Fraction	Area Fraction	Estimated Area (µm²)	Gundersen Error m=1
AONb	3	4	50	5	22500	202500	20	51	30	0.11	0.0158	108000.00	0.10
	4	5	50	5	22500	62500	20	131	33	0.36	0.0078	36666.7	0.08
	5	4	50	5	22500	62500	20	81	33	0.36	0.0135	36666.7	0.10
	6	3	50	5	22500	122500	10	62	42	0.18	0.0049	22866.70	0.09
	7	5	50	5	22500	90000	20	123	401	0.25	0.0153	105600.00	0.07
	8	2	50	5	22500	40000	10	166	625	0.56	0.0040	15466.70	0.09
	9	3	50	5	22500	160000	10	41	25	0.14	0.0050	17777.80	0.10
AONrb	18	3	50	7	40000	160000	20	42	29	0.25	0.0113	-	-
	19	4	50	7	40000	90000	20	130	54	0.44	0.0059	-	-
	24	2	50	7	40000	62500	20	124	51	0.64	0.0059	-	-
AONca	18	4	50	7	40000	62500	15	82	31	0.64	0.0044	-	-
	19	4	50	7	40000	62500	10	135	164	0.64	0.0051	-	-
	21	4	50	7	40000	62500	10	141	328	0.64	0.0090	-	-
	22	4	50	7	40000	62500	10	113	21	0.64	0.0008	-	-
	23	3	50	7	40000	62500	10	99	11	0.64	0.0005	-	-
AONcp	17	3	50	3	40000	62500	10	98	84	0.64	0.0038	-	-
	21	2	50	3	40000	62500	10	60	88	0.64	0.0063	-	-
	22	4	50	3	40000	62500	10	118	22	0.64	0.0008	-	-
	23	4	50	3	40000	62500	10	630	310	0.64	0.0066	-	-
	24	4	50	3	40000	62500	10	185	14	0.64	0.0003	-	-

Neuronal and glial characterization in the rostrocaudal axis of the human anterior olfactory nucleus: involvement in Parkinson's disease

Sandra Villar-Conde, Veronica Astillero-Lopez, Melania Gonzalez-Rodriguez, Daniel Saiz-Sanchez, Isabel Ubeda-Banon, Alicia Flores-Cuadrado*, Alino Martinez-Marcos*

Supplementary Table 9f. Area fraction occupied by total LNs.

	Case	Number of Sections	Section Cut Thickness (µm)	Section Evaluation Interval	Counting Frame Area (XY) (µm²)	Sampling Grid Area (XY) (µm²)	Grid Spacing (µm)	Number of Sampling Sites	Total Markers Counted	Area Sampling Fraction	Area Fraction	Estimated Area (µm²)	Gundersen Error m=1
AONb	3	4	50	5	22500	202500	20	51	30	0.11	0.0158	108000.00	0.10
	4	5	50	5	22500	62500	20	131	33	0.36	0.0078	36666.70	0.08
	5	4	50	5	22500	62500	20	81	33	0.36	0.0135	36666.70	0.10
	6	3	50	5	22500	122500	10	62	42	0.18	0.0049	22866.70	0.09
	7	5	50	5	22500	90000	20	123	401	0.25	0.0153	105600.00	0.07
	8	2	50	5	22500	40000	10	166	625	0.56	0.0040	15466.70	0.09
	9	3	50	5	22500	160000	10	41	25	0.14	0.0050	17777.80	0.10
AONrb	18	3	50	7	40000	160000	20	42	119	0.25	0.0462	-	-
	19	4	50	7	40000	90000	20	130	206	0.44	0.0224	-	-
	24	2	50	7	40000	62500	20	124	137	0.64	0.0160	-	-
AONca	18	4	50	7	40000	62500	15	82	60	0.64	0.0086	-	-
	19	4	50	7	40000	62500	10	135	446	0.64	0.0139	-	-
	21	4	50	7	40000	62500	10	141	1149	0.64	0.0315	-	-
	22	4	50	7	40000	62500	10	113	29	0.64	0.0011	-	-
	23	3	50	7	40000	62500	10	99	15	0.64	0.0007	-	-
AONcp	17	3	50	3	40000	62500	10	98	337	0.64	0.0151	-	-
	21	2	50	3	40000	62500	10	60	270	0.64	0.0193	-	-
	22	4	50	3	40000	62500	10	118	64	0.64	0.0022	-	-
	23	4	50	3	40000	62500	10	630	578	0.64	0.0123	-	-
	24	4	50	3	40000	62500	10	185	33	0.64	0.0008	-	-

Neuronal and glial characterization in the rostrocaudal axis of the human anterior olfactory nucleus: involvement in Parkinson's disease
Sandra Villar-Conde, Veronica Astillero-Lopez, Melania Gonzalez-Rodriguez, Daniel Saiz-Sanchez, Isabel Ubeda-Banon, Alicia Flores-Cuadrado*, Alino Martinez-Marcos*

Supplementary Table 10. Area fraction.

Supplementary Table 10a. Area fraction occupied by Iba-1 without overlapping.

	Case	Number of Sections	Section Cut Thickness (μm)	Section Evaluation Interval	Counting Frame Area (XY) (μm²)	Sampling Grid Area (XY) (μm²)	Grid Spacing (μm)	Number of Sampling Sites	Total Markers Counted	Area Sampling Fraction	Area Fraction	Estimated Area (μm²)	Gundersen Error m=1
AONrb	17	2	50	7	40000	62500	20	35	310	0.64	0.1462	193750.00	0.05
	18	3	50	7	40000	90000	20	74	528	0.44	0.1015	475200.00	0.03
	19	4	50	7	40000	90000	20	124	976	0.44	0.1174	878400.00	0.03
AONca	18	2	50	7	40000	62500	20	54	364	0.64	0.1303	227500.00	0.05
	19	2	50	7	40000	62500	20	93	759	0.64	0.1271	474375.00	0.11
	21	4	50	7	40000	90000	20	102	419	0.44	0.0686	377100.00	0.02
	23	3	50	7	40000	90000	20	70	359	0.44	0.0897	323100.00	0.02
AONcp	17	2	50	3	40000	62500	10	68	961	0.64	0.0551	150156.00	0.05
	21	3	50	3	40000	62500	10	78	1142	0.64	0.0542	178438.00	0.04
	22	4	50	3	40000	90000	10	84	814	0.44	0.0458	183150.00	0.02
	23	2	50	3	40000	62500	10	41	431	0.64	0.0481	67343.80	0.06
	24	2	50	3	40000	62500	10	72	96	0.64	0.0059	15000.00	0.06

Supplementary Table 10b. Area fraction occupied by LBs without overlapping.

	Case	Number of Sections	Section Cut Thickness (μm)	Section Evaluation Interval	Counting Frame Area (XY) (μm²)	Sampling Grid Area (XY) (μm²)	Grid Spacing (μm)	Number of Sampling Sites	Total Markers Counted	Area Sampling Fraction	Area Fraction	Estimated Area (μm²)	Gundersen Error m=1
AONrb	17	2	50	7	40000	62500	20	35	7	0.64	0.0033	4375.00	0.16
	18	3	50	7	40000	90000	20	74	19	0.44	0.0037	17100.00	0.10
	19	4	50	7	40000	90000	20	124	33	0.44	0.0040	29700.00	0.06
AONca	18	2	50	7	40000	62500	20	54	8	0.64	0.0029	5000.00	0.15
	19	2	50	7	40000	62500	20	93	21	0.64	0.0035	13125.00	0.11
	21	4	50	7	40000	90000	20	102	28	0.44	0.0046	25200.00	0.08
	23	3	50	7	40000	90000	20	70	5	0.44	0.0012	4500.00	0.26
AONcp	17	2	50	3	40000	62500	10	68	17	0.64	0.0010	2656.25	0.09
	21	3	50	3	40000	62500	10	78	51	0.64	0.0024	7968.75	0.05
	22	4	50	3	40000	90000	10	84	2	0.44	0.0001	450.00	0.53
	23	2	50	3	40000	62500	10	41	7	0.64	0.0008	1093.75	0.16
	24	2	50	3	40000	62500	10	72	1	0.64	0.0001	156.25	0.65

Neuronal and glial characterization in the rostrocaudal axis of the human anterior olfactory nucleus: involvement in Parkinson's disease

Sandra Villar-Conde, Veronica Astillero-Lopez, Melania Gonzalez-Rodriguez, Daniel Saiz-Sanchez, Isabel Ubeda-Banon, Alicia Flores-Cuadrado*, Alino Martinez-Marcos*

Supplementary Table 10c. Area fraction occupied by LNs without overlapping.

	Case	Number of Sections	Section Cut Thickness (μm)	Section Evaluation Interval	Counting Frame Area (XY) (μm²)	Sampling Grid Area (XY) (μm²)	Grid Spacing (μm)	Number of Sampling Sites	Total Markers Counted	Area Sampling Fraction	Area Fraction	Estimated Area (μm²)	Gundersen Error m=1
AONrb	17	2	50	7	40000	62500	20	35	551	0.64	0.0240	31875.00	0.06
	18	3	50	7	40000	90000	20	74	90	0.44	0.0173	81000.00	0.05
	19	4	50	7	40000	90000	20	124	143	0.44	0.0172	128700.00	0.03
AONca	18	2	50	7	40000	62500	20	54	41	0.64	0.0147	25625.00	0.08
	19	2	50	7	40000	62500	20	93	81	0.64	0.0136	50625.00	0.09
	21	4	50	7	40000	90000	20	102	233	0.44	0.0381	209700.00	0.03
	23	3	50	7	40000	90000	20	70	2	0.44	0.0005	1800.00	0.50
AONcp	17	2	50	3	40000	62500	10	68	97	0.64	0.0056	15156.30	0.05
	21	3	50	3	40000	62500	10	78	357	0.64	0.0169	55781.30	0.04
	22	4	50	3	40000	90000	10	84	41	0.44	0.0023	9225.00	0.06
	23	2	50	3	40000	62500	10	41	3	0.64	0.0003	468.75	0.28
	24	2	50	3	40000	62500	10	72	-	0.64	-	-	-

Supplementary Table 10d. Area fraction occupied by Iba-1 overlapping with LBs.

	Case	Number of Sections	Section Cut Thickness (μm)	Section Evaluation Interval	Counting Frame Area (XY) (μm²)	Sampling Grid Area (XY) (μm²)	Grid Spacing (μm)	Number of Sampling Sites	Total Markers Counted	Area Sampling Fraction	Area Fraction	Estimated Area (μm²)	Gundersen Error m=1
AONrb	17	2	50	7	40000	62500	20	35	3	0.64	0.0014	1875.00	0.28
	18	3	50	7	40000	90000	20	74	4	0.44	0.0008	3600.00	0.29
	19	4	50	7	40000	90000	20	124	1	0.44	0.0001	900.00	0.78
AONca	18	2	50	7	40000	62500	20	54	1	0.64	0.0004	625.00	0.65
	19	2	50	7	40000	62500	20	93	1	0.64	0.0002	625.00	0.11
	21	4	50	7	40000	90000	20	102	-	0.44	-	-	-
	23	3	50	7	40000	90000	20	70	-	0.44	-	-	-
AONcp	17	2	50	3	40000	62500	10	68	-	0.64	-	-	-
	21	3	50	3	40000	62500	10	78	2	0.64	0.0001	312.50	0.41
	22	4	50	3	40000	90000	10	84	-	0.44	-	-	-
	23	2	50	3	40000	62500	10	41	-	0.64	-	-	-
	24	2	50	3	40000	62500	10	72	-	0.64	-	-	-

Neuronal and glial characterization in the rostrocaudal axis of the human anterior olfactory nucleus: involvement in Parkinson's disease
Sandra Villar-Conde, Veronica Astillero-Lopez, Melania Gonzalez-Rodriguez, Daniel Saiz-Sanchez, Isabel Ubeda-Banon, Alicia Flores-Cuadrado*, Alino Martinez-Marcos*

Supplementary Table 10e. Area fraction occupied by Iba-1 overlapping with LNs.

	Case	Number of Sections	Section Cut Thickness (µm)	Section Evaluation Interval	Counting Frame Area (XY) (µm²)	Sampling Grid Area (XY) (µm²)	Grid Spacing (µm)	Number of Sampling Sites	Total Markers Counted	Area Sampling Fraction	Area Fraction	Estimated Area (µm²)	Gundersen Error m=1
AONrb	17	2	50	7	40000	62500	20	35	21	0.64	0.0099	13125.00	0.08
	18	3	50	7	40000	90000	20	74	3	0.44	0.0006	2700.00	0.03
	19	4	50	7	40000	90000	20	124	17	0.44	0.0020	15300.00	0.10
AONca	18	2	50	7	40000	62500	20	54	5	0.64	0.0018	3125.00	0.20
	19	2	50	7	40000	62500	20	93	10	0.64	0.0017	6250.00	0.39
	21	4	50	7	40000	90000	20	102	16	0.44	0.0026	14400.00	0.12
	23	3	50	7	40000	90000	20	70	-	0.44	-	-	-
AONcp	17	2	50	3	40000	62500	10	68	1	0.64	0.0001	156.25	0.11
	21	3	50	3	40000	62500	10	78	9	0.64	0.0004	1406.25	0.14
	22	4	50	3	40000	90000	10	84	13	0.44	0.0007	2925.00	0.16
	23	2	50	3	40000	62500	10	41	-	0.64	-	-	-
	24	2	50	3	40000	62500	10	72	-	0.64	-	-	-

Supplementary Table 10f. Area fraction occupied by total Iba-1.

	Case	Number of Sections	Section Cut Thickness (µm)	Section Evaluation Interval	Counting Frame Area (XY) (µm²)	Sampling Grid Area (XY) (µm²)	Grid Spacing (µm)	Number of Sampling Sites	Total Markers Counted	Area Sampling Fraction	Area Fraction	Estimated Area (µm²)	Gundersen Error m=1
AONrb	17	2	50	7	40000	62500	20	35	334	0.64	0.1575	-	-
	18	3	50	7	40000	90000	20	74	535	0.44	0.1029	-	-
	19	4	50	7	40000	90000	20	124	994	0.44	0.1196	-	-
AONca	18	2	50	7	40000	62500	20	54	369	0.64	0.1321	-	-
	19	2	50	7	40000	62500	20	93	770	0.64	0.1289	-	-
	21	4	50	7	40000	90000	20	102	435	0.44	0.0712	-	-
	23	3	50	7	40000	90000	20	70	359	0.44	0.0897	-	-
AONcp	17	2	50	3	40000	62500	10	68	962	0.64	0.0552	-	-
	21	3	50	3	40000	62500	10	78	1153	0.64	0.0548	-	-
	22	4	50	3	40000	90000	10	84	827	0.44	0.0481	-	-
	23	2	50	3	40000	62500	10	72	96	0.64	0.0059	-	-
	24	2	50	3	40000	62500	10	41	431	0.64	0.0481	-	-

Neuronal and glial characterization in the rostrocaudal axis of the human anterior olfactory nucleus: involvement in Parkinson's disease

Sandra Villar-Conde, Veronica Astillero-Lopez, Melania Gonzalez-Rodriguez, Daniel Saiz-Sanchez, Isabel Ubeda-Banon, Alicia Flores-Cuadrado*, Alino Martinez-Marcos*

Supplementary Table 10g. Area fraction occupied by total LBs.

	Case	Number of Sections	Section Cut Thickness (µm)	Section Evaluation Interval	Counting Frame Area (XY) (µm²)	Sampling Grid Area (XY) (µm²)	Grid Spacing (µm)	Number of Sampling Sites	Total Markers Counted	Area Sampling Fraction	Area Fraction	Estimated Area (µm²)	Gundersen Error m=1
AONrb	17	2	50	7	40000	62500	20	35	10	0.64	0.0047	-	-
	18	3	50	7	40000	90000	20	74	25	0.44	0.0048	-	-
	19	4	50	7	40000	90000	20	124	34	0.44	0.0041	-	-
AONca	18	2	50	7	40000	62500	20	54	9	0.64	0.0032	-	-
	19	2	50	7	40000	62500	20	93	22	0.64	0.0037	-	-
	21	4	50	7	40000	90000	20	102	28	0.44	0.0046	-	-
	23	3	50	7	40000	90000	20	70	5	0.44	0.0012	-	-
AONcp	17	2	50	3	40000	62500	10	68	17	0.64	0.0010	-	-
	21	3	50	3	40000	62500	10	78	53	0.64	0.0025	-	-
	22	4	50	3	40000	90000	10	84	2	0.44	0.0001	-	-
	23	2	50	3	40000	62500	10	72	1	0.64	0.0001	-	-
	24	2	50	3	40000	62500	10	41	7	0.64	0.0008	-	-

Supplementary Table 10h. Area fraction occupied by total LNs.

	Case	Number of Sections	Section Cut Thickness (µm)	Section Evaluation Interval	Counting Frame Area (XY) (µm²)	Sampling Grid Area (XY) (µm²)	Grid Spacing (µm)	Number of Sampling Sites	Total Markers Counted	Area Sampling Fraction	Area Fraction	Estimated Area (µm²)	Gundersen Error m=1
AONrb	17	2	50	7	40000	62500	20	35	72	0.64	0.0339	-	-
	18	3	50	7	40000	90000	20	74	93	0.44	0.0179	-	-
	19	4	50	7	40000	90000	20	124	160	0.44	0.0193	-	-
AONca	18	2	50	7	40000	62500	20	54	46	0.64	0.0165	-	-
	19	2	50	7	40000	62500	20	93	91	0.64	0.0152	-	-
	21	4	50	7	40000	90000	20	102	249	0.44	0.0407	-	-
	23	3	50	7	40000	90000	20	70	2	0.44	0.0005	-	-
AONcp	17	2	50	3	40000	62500	10	68	98	0.64	0.0056	-	-
	21	3	50	3	40000	62500	10	78	366	0.64	0.0174	-	-
	22	4	50	3	40000	90000	10	84	54	0.44	0.0031	-	-
	23	2	50	3	40000	62500	10	72	0	0.64	0.0000	-	-
	24	2	50	3	40000	62500	10	41	3	0.64	0.0003	-	-

Neuronal and glial characterization in the rostrocaudal axis of the human anterior olfactory nucleus: involvement in Parkinson's disease

Sandra Villar-Conde, Veronica Astillero-Lopez, Melania Gonzalez-Rodriguez, Daniel Saiz-Sanchez, Isabel Ubeda-Banon, Alicia Flores-Cuadrado*, Alino Martinez-Marcos*

Supplementary Table 11. Area fraction.

Supplementary Table 11a. Area fraction occupied by GFAP without overlapping.

	Case	Number of Sections	Section Cut Thickness (µm)	Section Evaluation Interval	Counting Frame Area (XY) (µm²)	Sampling Grid Area (XY) (µm²)	Grid Spacing (µm)	Number of Sampling Sites	Total Markers Counted	Area Sampling Fraction	Area Fraction	Estimated Area (µm²)	Gundersen Error m=1
AONb	14	3	50	5	40000	62500	20	68	643	0.64	0.1596	401875.00	0.02
	13	2	50	5	40000	62500	20	50	223	0.64	0.1016	139375.00	0.11
	12	5	50	5	40000	62500	20	143	1019	0.64	0.1521	636875.00	0.04
	11	3	50	5	40000	62500	20	98	771	0.64	0.1405	481875.00	0.03
	15	4	50	5	40000	62500	20	82	606	0.64	0.1584	378750.00	0.03
AONrb	17	2	50	7	40000	62500	20	46	323	0.64	0.1407	201875.00	0.05
	18	3	50	7	40000	90000	20	59	456	0.44	0.1281	410400.00	0.04
	19	2	50	7	40000	62500	20	84	837	0.64	0.1452	523125.00	0.05
	20	4	50	7	40000	90000	20	100	944	0.44	0.1546	849600.00	0.03
AONca	18	2	50	7	40000	62500	20	46	539	0.64	0.2214	336875.00	0.05
	19	4	50	7	40000	90000	20	93	974	0.44	0.1908	876600.00	0.02
	21	4	50	7	40000	90000	20	114	864	0.44	0.1165	777600.00	0.02
	22	4	50	7	40000	90000	20	92	887	0.44	0.0002	900.00	0.88
AONcp	17	3	50	3	40000	90000	10	75	1872	0.44	0.1016	421200.00	0.02
	22	4	50	3	40000	90000	10	68	1301	0.44	0.0825	292725.00	0.02
	23	3	50	3	40000	90000	10	73	1591	0.44	0.0822	357975.00	0.02

Neuronal and glial characterization in the rostrocaudal axis of the human anterior olfactory nucleus: involvement in Parkinson's disease

Sandra Villar-Conde, Veronica Astillero-Lopez, Melania Gonzalez-Rodriguez, Daniel Saiz-Sanchez, Isabel Ubeda-Banon, Alicia Flores-Cuadrado*, Alino Martinez-Marcos*

Supplementary Table 11b. Area fraction occupied by LBs without overlapping.

	Case	Number of Sections	Section Cut Thickness (µm)	Section Evaluation Interval	Counting Frame Area (XY) (µm²)	Sampling Grid Area (XY) (µm²)	Grid Spacing (µm)	Number of Sampling Sites	Total Markers Counted	Area Sampling Fraction	Area Fraction	Estimated Area (µm²)	Gundersen Error m=1
AONb	14	3	50	5	40000	62500	20	68	2	0.64	0.0005	1250.00	0.46
	13	2	50	5	40000	62500	20	50	5	0.64	0.0023	3125.00	0.22
	12	5	50	5	40000	62500	20	143	9	0.64	0.0013	5625.00	0.18
	11	3	50	5	40000	62500	20	98	6	0.64	0.0011	3750.00	0.25
	15	4	50	5	40000	62500	20	82	2	0.64	0.0005	1250.00	0.57
AONrb	17	2	50	7	40000	62500	20	46	6	0.64	0.0026	3750.00	0.18
	18	3	50	7	40000	90000	20	59	6	0.44	0.0017	5400.00	0.20
	19	2	50	7	40000	62500	20	84	25	0.64	0.0043	15625.00	0.08
	20	4	50	7	40000	90000	20	100	23	0.44	0.0038	20700.00	0.09
AONca	18	2	50	7	40000	62500	20	46.00	4	0.64	0.0016	2500.00	0.23
	19	4	50	7	40000	90000	20	93	16	0.44	0.0031	14400.00	0.12
	21	4	50	7	40000	90000	20	114	29	0.44	0.0039	26100.00	0.08
	22	4	50	7	40000	90000	20	92	2	0.44	0.0004	1800.00	0.52
AONcp	17	3	50	3	40000	90000	10	75	22	0.44	0.0012	4950.00	0.09
	22	4	50	3	40000	90000	10	68	2	0.44	0.0001	450.00	0.54
	23	3	50	3	40000	90000	10	73	17	0.44	0.0009	3825.00	0.10

Neuronal and glial characterization in the rostrocaudal axis of the human anterior olfactory nucleus: involvement in Parkinson's disease

Sandra Villar-Conde, Veronica Astillero-Lopez, Melania Gonzalez-Rodriguez, Daniel Saiz-Sanchez, Isabel Ubeda-Banon, Alicia Flores-Cuadrado*, Alino Martinez-Marcos*

Supplementary Table 11c. Area fraction occupied by LNs without overlapping.

	Case	Number of Sections	Section Cut Thickness (µm)	Section Evaluation Interval	Counting Frame Area (XY) (µm²)	Sampling Grid Area (XY) (µm²)	Grid Spacing (µm)	Number of Sampling Sites	Total Markers Counted	Area Sampling Fraction	Area Fraction	Estimated Area (µm²)	Gundersen Error m=1
AONb	14	3	50	5	40000	62500	20	68	60	0.64	0.0149	37500.00	0.04
	13	2	50	5	40000	62500	20	50	117	0.64	0.0533	73125.00	0.11
	12	5	50	5	40000	62500	20	143	227	0.64	0.0339	141875.00	0.04
	11	3	50	5	40000	62500	20	98	137	0.64	0.0250	85625.00	0.04
	15	4	50	5	40000	62500	20	82	149	0.64	0.0390	93125.00	0.04
AONrb	17	2	50	7	40000	62500	20	46	46	0.64	0.0200	28750.00	0.07
	18	3	50	7	40000	90000	20	59	40	0.44	0.0112	36000.00	0.07
	19	2	50	7	40000	62500	20	84	148	0.64	0.0257	92500.00	0.05
	20	4	50	7	40000	90000	20	100	34	0.44	0.0056	30600.00	0.08
AONca	18	2	50	7	40000	62500	20	46	33	0.64	0.0136	20625.00	0.07
	19	4	50	7	40000	90000	20	93	107	0.44	0.0210	96300.00	0.04
	21	4	50	7	40000	90000	20	114	327	0.44	0.0441	294300.00	0.02
	22	4	50	7	40000	90000	20	92	13	0.44	0.0023	11700.00	0.13
AONcp	17	3	50	3	40000	90000	10	75	308	0.44	0.0167	69300.00	0.04
	22	4	50	3	40000	90000	10	68	97	0.44	0.0061	21825.00	0.04
	23	3	50	3	40000	90000	10	73	325	0.44	0.0168	73125.00	0.03

Neuronal and glial characterization in the rostrocaudal axis of the human anterior olfactory nucleus: involvement in Parkinson's disease

Sandra Villar-Conde, Veronica Astillero-Lopez, Melania Gonzalez-Rodriguez, Daniel Saiz-Sanchez, Isabel Ubeda-Banon, Alicia Flores-Cuadrado*, Alino Martinez-Marcos*

Supplementary Table 11d. Area fraction occupied by GFAP overlapping with LBs.

	Case	Number of Sections	Section Cut Thickness (µm)	Section Evaluation Interval	Counting Frame Area (XY) (µm²)	Sampling Grid Area (XY) (µm²)	Grid Spacing (µm)	Number of Sampling Sites	Total Markers Counted	Area Sampling Fraction	Area Fraction	Estimated Area (µm²)	Gundersen Error m=1
AONb	14	3	50	5	40000	62500	20	68	2	0.64	0.0005	1250.00	0.47
	13	2	50	5	40000	62500	20	50	-	0.64	-	-	-
	12	5	50	5	40000	62500	20	143	-	0.64	-	-	-
	11	3	50	5	40000	62500	20	98	1	0.64	0.0002	625.00	0.93
	15	4	50	5	40000	62500	20	82	2	0.64	0.0005	1250.00	0.57
AONrb	17	2	50	7	40000	62500	20	46	1	0.64	0.0004	625.00	0.65
	18	3	50	7	40000	90000	20	59	-	0.44	-	-	-
	19	2	50	7	40000	62500	20	84	4	0.64	0.0007	2500.00	0.25
	20	4	50	7	40000	90000	20	100	-	0.44	-	-	-
AONca	18	2	50	7	40000	62500	20	46	-	0.64	-	-	-
	19	4	50	7	40000	90000	20	93	1	0.44	0.0002	900.00	0.89
	21	4	50	7	40000	90000	20	114	-	0.44	-	-	-
	22	4	50	7	40000	90000	20	92	-	0.44	-	-	-
AONcp	17	3	50	3	40000	90000	10	75	-	0.44	-	-	-
	22	4	50	3	40000	90000	10	68	-	0.44	-	-	-
	23	3	50	3	40000	90000	10	73	-	0.44	-	-	-

Neuronal and glial characterization in the rostrocaudal axis of the human anterior olfactory nucleus: involvement in Parkinson's disease

Sandra Villar-Conde, Veronica Astillero-Lopez, Melania Gonzalez-Rodriguez, Daniel Saiz-Sanchez, Isabel Ubeda-Banon, Alicia Flores-Cuadrado*, Alino Martinez-Marcos*

Supplementary Table 11e. Area fraction occupied by GFAP overlapping with LNs.

	Case	Number of Sections	Section Cut Thickness (µm)	Section Evaluation Interval	Counting Frame Area (XY) (µm²)	Sampling Grid Area (XY) (µm²)	Grid Spacing (µm)	Number of Sampling Sites	Total Markers Counted	Area Sampling Fraction	Area Fraction	Estimated Area (µm²)	Gundersen Error m=1
AONb	14	3	50	5	40000	62500	20	68	-	0.64	-	-	-
	13	2	50	5	40000	62500	20	50	6	0.64	0.0027	3750.00	0.39
	12	5	50	5	40000	62500	20	143	14	0.64	0.0021	8750.00	0.14
	11	3	50	5	40000	62500	20	98	3	0.64	0.0005	1875.00	0.41
	15	4	50	5	40000	62500	20	82	13	0.64	0.0034	8125.00	0.15
AONrb	17	2	50	7	40000	62500	20	46	7	0.64	0.0031	4375.00	0.16
	18	3	50	7	40000	90000	20	59	6	0.44	0.0017	5400.00	0.20
	19	2	50	7	40000	62500	20	84	17	0.64	0.0029	10625.00	0.11
	20	4	50	7	40000	90000	20	100	2	0.44	0.0003	1800.00	0.55
AONca	18	2	50	7	40000	62500	20	46	1	0.64	0.0004	625.00	0.65
	19	4	50	7	40000	90000	20	93	15	0.44	0.0029	13500.00	0.12
	21	4	50	7	40000	90000	20	114	25	0.44	0.0034	22500.00	0.09
	22	4	50	7	40000	90000	20	92	1	0.44	0.0002	900.00	0.88
AONcp	17	3	50	3	40000	90000	10	75	23	0.44	0.0012	5175.00	0.10
	22	4	50	3	40000	90000	10	68	2	0.44	0.0001	450.00	0.54
	23	3	50	3	40000	90000	10	73	12	0.44	0.0006	2700.00	0.14

Neuronal and glial characterization in the rostrocaudal axis of the human anterior olfactory nucleus: involvement in Parkinson's disease

Sandra Villar-Conde, Veronica Astillero-Lopez, Melania Gonzalez-Rodriguez, Daniel Saiz-Sanchez, Isabel Ubeda-Banon, Alicia Flores-Cuadrado*, Alino Martinez-Marcos*

Supplementary Table 11f. Area fraction occupied by total GFAP.

	Case	Number of Sections	Section Cut Thickness (µm)	Section Evaluation Interval	Counting Frame Area (XY) (µm²)	Sampling Grid Area (XY) (µm²)	Grid Spacing (µm)	Number of Sampling Sites	Total Markers Counted	Area Sampling Fraction	Area Fraction	Estimated Area (µm²)	Gundersen Error m=1
AONb	14	3	50	5	40000	62500	20	68	645	0.64	0.1601	-	-
	13	2	50	5	40000	62500	20	50	229	0.64	0.1043	-	-
	12	5	50	5	40000	62500	20	143	1033	0.64	0.1542	-	-
	11	3	50	5	40000	62500	20	98	774	0.64	0.1411	-	-
	15	4	50	5	40000	62500	20	82	621	0.64	0.1624	-	-
AONrb	17	2	50	7	40000	62500	20	46	330	0.64	0.1438	-	-
	18	3	50	7	40000	90000	20	59	462	0.44	0.1298	-	-
	19	2	50	7	40000	62500	20	84	858	0.64	0.1488	-	-
	20	4	50	7	40000	90000	20	100	946	0.44	0.1549	-	-
AONca	18	2	50	7	40000	62500	20	46	540	0.64	0.2218	-	-
	19	4	50	7	40000	90000	20	93	990	0.44	0.1940	-	-
	21	4	50	7	40000	90000	20	114	889	0.44	0.1198	-	-
	22	4	50	7	40000	90000	20	92	888	0.44	0.1562	-	-
AONcp	17	3	50	3	40000	90000	10	75	1895	0.44	0.1029	-	-
	22	4	50	3	40000	90000	10	68	1303	0.44	0.0826	-	-
	23	3	50	3	40000	90000	10	73	1603	0.44	0.0828	-	-

Neuronal and glial characterization in the rostrocaudal axis of the human anterior olfactory nucleus: involvement in Parkinson's disease

Sandra Villar-Conde, Veronica Astillero-Lopez, Melania Gonzalez-Rodriguez, Daniel Saiz-Sanchez, Isabel Ubeda-Banon, Alicia Flores-Cuadrado*, Alino Martinez-Marcos*

Supplementary Table 11g. Area fraction occupied by total LB.

	Case	Number of Sections	Section Cut Thickness (µm)	Section Evaluation Interval	Counting Frame Area (XY) (µm²)	Sampling Grid Area (XY) (µm²)	Grid Spacing (µm)	Number of Sampling Sites	Total Markers Counted	Area Sampling Fraction	Area Fraction	Estimated Area (µm²)	Gundersen Error m=1
AONb	14	3	50	5	40000	62500	20	68	4	0.64	0.0010	-	-
	13	2	50	5	40000	62500	20	50	5	0.64	0.0023	-	-
	12	5	50	5	40000	62500	20	143	9	0.64	0.0013	-	-
	11	3	50	5	40000	62500	20	98	7	0.64	0.0013	-	-
	15	4	50	5	40000	62500	20	82	4	0.64	0.0010	-	-
AONrb	17	2	50	7	40000	62500	20	46	7	0.64	0.0031	-	-
	18	3	50	7	40000	90000	20	53	6	0.44	0.0017	-	-
	19	2	50	7	40000	62500	20	84	29	0.44	0.0050	-	-
	20	4	50	7	40000	90000	20	100	23	0.44	0.0038	-	-
AONca	18	2	50	7	40000	62500	20	46	4	0.64	0.0016	-	-
	19	4	50	7	40000	90000	20	93	17	0.44	0.0033	-	-
	21	4	50	7	40000	90000	20	114	29	0.44	0.0039	-	-
	22	4	50	7	40000	90000	20	92	2	0.44	0.0004	-	-
AONcp	17	3	50	3	40000	90000	10	75	22	0.44	0.0012	-	-
	22	4	50	3	40000	90000	10	68	2	0.44	0.0001	-	-
	23	3	50	3	40000	90000	10	73	17	0.44	0.0009	-	-

Neuronal and glial characterization in the rostrocaudal axis of the human anterior olfactory nucleus: involvement in Parkinson's disease
Sandra Villar-Conde, Veronica Astillero-Lopez, Melania Gonzalez-Rodriguez, Daniel Saiz-Sanchez, Isabel Ubeda-Banon, Alicia Flores-Cuadrado*, Alino Martinez-Marcos*

Supplementary Table 11h. Area fraction occupied by total LN.

	Case	Number of Sections	Section Cut Thickness (μm)	Section Evaluation Interval	Counting Frame Area (XY) (μm²)	Sampling Grid Area (XY) (μm²)	Grid Spacing (μm)	Number of Sampling Sites	Total Markers Counted	Area Sampling Fraction	Area Fraction	Estimated Area (μm²)	Gundersen Error m=1
AONb	14	3	50	5	40000	62500	20	68	60	0.64	0.0149	-	-
	13	2	50	5	40000	62500	20	50	123	0.64	0.0560	-	-
	12	5	50	5	40000	62500	20	143	241	0.64	0.0360	-	-
	11	3	50	5	40000	62500	20	98	140	0.64	0.0255	-	-
	15	4	50	5	40000	62500	20	82	162	0.64	0.0424	-	-
AONrb	17	2	50	7	40000	62500	20	46	53	0.64	0.0231	-	-
	18	3	50	7	40000	90000	20	53	46	0.44	0.0129	-	-
	19	2	50	7	40000	62500	20	84	5	0.44	0.0009	-	-
	20	4	50	7	40000	90000	20	100	36	0.44	0.0059	-	-
AONca	18	2	50	7	40000	62500	20	46	34	0.64	0.0140	-	-
	19	4	50	7	40000	90000	20	93	25	0.44	0.0049	-	-
	21	4	50	7	40000	90000	20	114	352	0.44	0.0475	-	-
	22	4	50	7	40000	90000	20	92	14	0.44	0.0025	-	-
AONcp	17	3	50	3	40000	90000	10	75	331	0.44	0.0180	-	-
	22	4	50	3	40000	90000	10	68	99	0.44	0.0063	-	-
	23	3	50	3	40000	90000	10	73	337	0.44	0.0174	-	-
